# Review on Experimental and Theoretical Investigations of Ultra-Short Pulsed Laser Ablation of Metals with Burst Pulses

**DOI:** 10.3390/ma14123331

**Published:** 2021-06-16

**Authors:** Daniel J. Förster, Beat Jäggi, Andreas Michalowski, Beat Neuenschwander

**Affiliations:** 1 Institut für Strahlwerkzeuge, University of Stuttgart, Pfaffenwaldring 43, 70569 Stuttgart, Germany; 2 LightPulse LASER PRECISION, Pfaffenwaldring 43, 70569 Stuttgart, Germany; 3 LASEA Switzerland SA, Rue du Soleil 11, 2504 Bienne, Switzerland; bjaeggi@lasea.com; 4 Robert Bosch GmbH, Renningen, 70465 Stuttgart, Germany; andreas.michalowski@de.bosch.com; 5 Institute for Applied Laser, Photonics and Surface Technologies ALPS, Bern University of Applied Sciences, Pestalozzistrasse 20, 3400 Burgdorf, Switzerland

**Keywords:** laser ablation, ultra-short pulses, double pulses, burst processing, MHz bursts, GHz bursts

## Abstract

Laser processing with ultra-short double pulses has gained attraction since the beginning of the 2000s. In the last decade, pulse bursts consisting of multiple pulses with a delay of several 10 ns and less found their way into the area of micromachining of metals, opening up completely new process regimes and allowing an increase in the structuring rates and surface quality of machined samples. Several physical effects such as shielding or re-deposition of material have led to a new understanding of the related machining strategies and processing regimes. Results of both experimental and numerical investigations are placed into context for different time scales during laser processing. This review is dedicated to the fundamental physical phenomena taking place during burst processing and their respective effects on machining results of metals in the ultra-short pulse regime for delays ranging from several 100 fs to several microseconds. Furthermore, technical applications based on these effects are reviewed.

## 1. Introduction

To enable increased throughput, the development of ultra-short pulsed lasers for materials processing has always aimed for higher laser powers. In recent years, ultrafast lasers with average output powers exceeding 1 kW [1,2,3,4,5] and even 10 kW [6] have been demonstrated. Industrially available lasers currently already provide pulse energies of up to some 100 μJ, whereas laser systems in academia provide several mJ. Thus, the available energy per pulse is often much higher than the energy actually needed for single pulse ablation. In order to attain the highest possible ablated volume for a given amount of energy, the energy can be distributed among pulses within a pulse package, a so-called pulse burst consisting of a minimum of two pulses and up to several hundred or thousand pulses [7,8,9,10,11,12,13,14,15,16,17,18,19,20,21,22,23,24]. Each of these burst packages then carries a sufficiently high amount of energy that can be used for laser machining, while the total energy of all pulses together is close to the maximum available pulse energy of the laser. In recent years, ultra-short pulsed laser ablation with burst pulses for micromachining has been intensively investigated both experimentally and numerically.

The aim of this article was to provide a comprehensive review of the physics of ultra-short pulsed laser ablation of metals with burst pulses and the applications based on the physical processes involved. In the next subsection, processing strategies and definitions used in this article are introduced. In the second section, the physics of ablation using ultra-short burst pulses of metals is discussed, summarizing the physics involved during single pulse laser ablation in the first place, followed by the pulse-to-pulse interactions of two subsequent ultra-short laser pulses. Afterwards, special attention is paid to machining with triple pulses, followed by two sections dedicated to the physics involved when processing with multiple pulses with intra-burst repetition rates of MHz and GHz. In Section 3, several MHz and GHz burst processing applications are summarized and reviewed, ranging from punching and drilling processes via line scribing, milling, and surface structuring to several special applications. The article closes with conclusions and an outlook.

### Processing Strategies and Definitions

**Figure 1:**
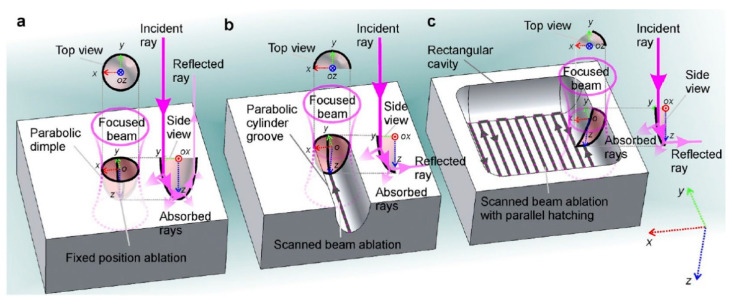
Schematic illustration of the processes and machining results: (**a**) dimple with the shape of a paraboloid ablated by multiple laser pulses with a stationary beam, resulting out of the punching process; (**b**) groove with the shape of a parabolic cylinder ablated by a scanned laser beam (line scan), representing the scribing process; (**c**) rectangular-shaped cavity ablated by a scanned laser beam and parallel line hatching (bidirectional meandering scan) resulting out of laser milling. The 3D Cartesian coordinate system x (red arrow), y (green arrow), and z (blue arrow) holds for all sketches (**a**–**c**). The center positions of the focused Gaussian beams are indicated by o. The multiple reflections of incident rays from the interior walls are given as side views (yz planes). Incident and multiple reflected rays are also given as side views (yz planes). The interaction area (cf. xy and yz planes) of laser and material decreases from (**a**–**c**). Reproduced under the terms of a Creative Commons Attribution 4.0 International License, (https://creativecommons.org/licenses/by/4.0/ (accessed on 31 May 2021)) [26]. Copyright 2018, the authors, published by Springer Nature.

In material processing, different strategies can be applied depending on the application. Using a stationary beam, the so-called punching process is realized. In this process, a certain number of consecutive laser pulses N is focused on the same spot and each pulse ablates a thin layer of material. Using a Gaussian beam with a $1/e^{2}$-radius of w, this results in a parabolic dimple, as shown in Figure 1a. If the applied number of pulses and the fluence are high enough, the material can be drilled through. This drilling strategy is called percussion drilling and represents only one of the possible drilling strategies used in ultra-short pulse laser machining [25].

In contrast to using a stationary beam, scribing can be realized if the laser beam is moved along one lateral direction. This results on the workpiece in a parabolic-shaped groove if a Gaussian beam is used (cf. Figure 1b). The linear movement of the beam can be realized using a linear stage or a galvanometric scanner for example. In the scribing process, consecutive pulses overlap each other. The overlap $o_{line}$ can be calculated using the moving speed of the axis $v_{scan}$, the radius w, and the used pulse repetition rate of the laser $f_{rep}$ by

(1) $$o_{line}=1-\frac{v_{scan}}{f_{rep}\cdot 2\cdot w}$$

If a deeper groove is demanded, the laser beam is moved several times along the same line path. These repetitions are the number of machined layers or overscans $N_{L}$. If the ratio between material thickness, laser parameters, and repetitions is chosen correctly, the material can even be cut through using this line scribing technique. The third ablation process, called milling (cf. Figure 1c), is realized by machining several parallel lines with a defined line spacing. This line spacing is called the hatch distance $d_{h}$. In the milling process, in addition to the overlap of two consecutive pulses along the scan direction, there is also an overlapping in the perpendicular direction to the machined lines $o_{cross}$:

(2) $$o_{cross}=1-\frac{d_{h}}{2\cdot w}$$

**Figure 2:**
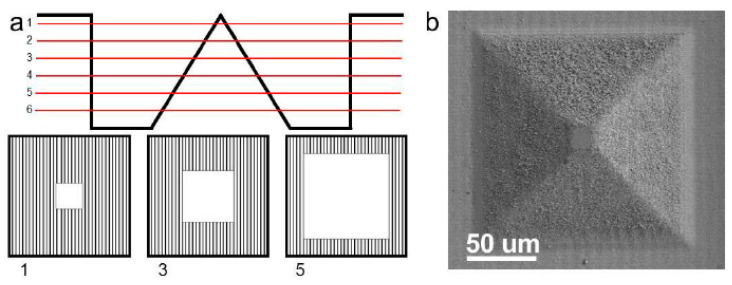
2.5D processing; (**a**) slicing of the 3D structure and three corresponding 2D slices with the hatch pattern (no rotation of the hatch pattern from layer to layer); (**b**) SEM image of a 3D structure machined in copper. Reprinted from [27], copyright 2013, with permission from Elsevier.

In milling applications, the laser spot must be moved in two lateral directions. The straight-forward approach is to use a galvanometric scanner to perform this movement [26]. Additionally, solutions using a rotational axis in combination with different technologies such as linear stages [27], galvanometric scanners [28], or acousto-optic deflectors (AODs) [29] are used to allow such 2D movement. In the z-direction, the material is removed layer by layer, resulting in a so-called 2.5D process [27]. Using this strategy, 3D structures can be engraved by changing the processing region from layer to layer, as shown in Figure 2a. In addition, the direction of the hatch pattern (direction of the parallel lines) can be rotated from layer to layer by the hatch angle to avoid scanning the same lines multiple times, which could lead to line irregularities. If a central unprocessed square in the middle of a process region is enlarged linearly layer by layer (making the scanned area per layer smaller), the result is a square pyramid as a subtractively generated geometry (cf. Figure 2b).

**Figure 3:**
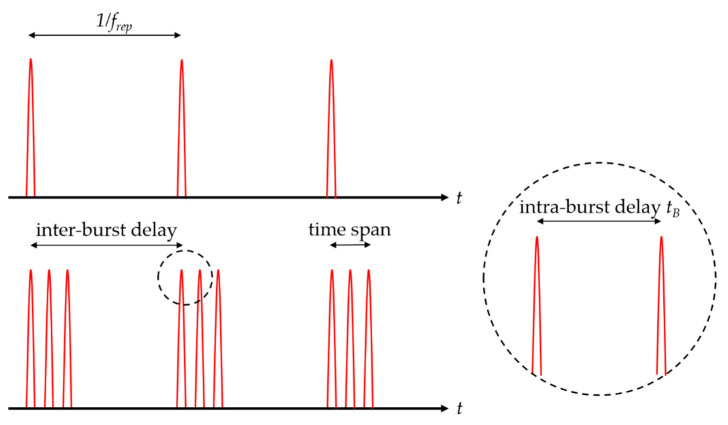
Upper row: single pulse operation with a defined repetition rate *frep*; lower row: burst operation with display of the two delay times inter-burst delay and intra-burst delay *t_B_* as well as the time span.

The described processing strategies have been used principally with single pulses, which are emitted at a given repetition rate from the used laser system. In burst processing, the single pulse is replaced by a pulse package including several pulses with a temporal distance much shorter than the pulse repetition rate (intra-burst delay $t_{B}$, cf. Figure 3). This package of pulses is called a burst. Depending on the intra-burst delay time, it can be distinguished between MHz bursts (with an intra-burst delay of tens of ns) and GHz bursts (with an intra-burst delay of hundreds of ps). The temporal distance between the burst packages is called the inter-burst delay and corresponds to the pulse repetition rate in single pulse operation. During scribing and milling, it can be assumed that for such time delays the burst package is applied within the area of one sole spot, i.e., it acts similarly to a stationary beam.

## 2. Physics of Ultra-Short Burst Pulse Ablation of Metals

The focus of this article lies on metal processing. For laser processing of different materials such as semiconductors [30,31,32], dielectrics [30,31,33,34,35], or ceramics [31,36], the reader may refer to the corresponding articles. In the following, the physical mechanisms of laser–material interactions relevant in the machining of metals are discussed. First, the mechanisms during and after the processing of metals with single ultra-short laser pulses (for repetitive processing, this corresponds to inter-pulse delays of tens of microseconds or repetition rates below 100 kHz) is discussed and summarized in Section 2.1. Although several articles exist on this topic and deal especially with the micromachining of different metals in this regime [30,31,37,38,39,40], a summary of the essential effects regarding processing in a single pulse operation is given for completion and as a basis for the following sections. After introducing and summarizing the involved physical mechanisms for single pulse laser ablation of metals, the pulse-to-pulse interactions of two subsequent pulses are introduced in Section 2.2. The focus in that section lies on interactions between two pulses with inter-pulse delays of several 100 fs to several 10 ns, since the experimental and theoretical findings differ fundamentally from metal processing in single pulse operation. In Section 2.3, the physical mechanisms and state-of-the art findings for triple pulse processing with intra-burst delays in the range of tens of ns are discussed. Finally, in Section 2.4 and Section 2.5, machining with bursts containing multiple pulses with intra-burst repetition rates of MHz and GHz are summarized.

### 2.1. Single Pulse Laser Ablation

When light irradiates a metal surface, photons interact with free electrons and energy is transferred to the electronic system via inverse Bremsstrahlung. Subsequently, the electronic system transfers energy to the lattice (ions) and excites vibrations of the ionic system, leading to a heating of the metal surface resulting in an increase of temperature. For laser pulses with pulse durations strongly exceeding the electron–phonon coupling time, the excitation of the electronic and ionic system can be considered to happen in thermal equilibrium. Hence, conventional heat conduction and physical mechanisms such as melting and evaporation take place. During ultra-short pulsed processing, short heating times allow physical states of matter to cross borders in the phase diagram (e.g., the solid–liquid line) within picoseconds and induce high pressures (tens of GPa and more) within tens of picoseconds within thin surface layers [41,42,43,44,45,46]. For a deeper understanding, details of the involved physical mechanisms can be found in several references including theoretical and numerical modeling [37,41,42,43,44,45,46]. The most important measure to distinguish between the ultra-short pulse regime and classical thermal heating in equilibrium is the electron–phonon coupling time $\tau_{el-phon}$, which is a characteristic time for heating the ionic lattice. In first approximation, this value is a constant, but, in principle, it depends on the deposited energy, and the time of relaxation can be multiples of that characteristic time [23]. It can be estimated by $\tau_{el-phon}=C_{P}/\gamma$, with $C_{P}$ being the heat capacity of the ionic system and γ being the electron-phonon coupling constant [37]. Both values are material-dependent. For metals and their alloys, the electron–phonon coupling time lies in the range of several 100 fs to more than 100 ps (e.g., pure gold even has a coupling time of around 115 ps). Exemplary values are given in Table 1. For alloys, the electron–phonon coupling times can differ from the original elements forming the alloy, e.g., for AISI 304 stainless steel, an electron–phonon coupling time of 0.5 ps was reported [47], whereas it amounts to 1.3 ps for pure iron.

Another crucial quantity for the description of different regimes of laser processing with ultra-short pulses is the laser fluence ϕ. It is defined as the energy per irradiated area, and for a Gaussian beam the peak fluence $\phi_{0}$ equals

(3) $$\phi_{0}=2\cdot \phi_{av}=2\cdot \frac{E_{P}}{\pi \cdot w^{2}}$$

$E_{P}$ corresponds to the pulse energy of the incoming laser pulse, w to the laser spot radius (1/e^2^), and $\phi_{av}$ is the average fluence. The fluence can be increased by increasing the laser pulse energy or decreasing the irradiated area (spot size). If the fluence is increased above the ablation threshold $\phi_{th}$, material is removed from the surface (ablation). With increasing fluence, the ultra-short pulsed laser interaction with metals can be characterized by the following physical processes [41,42,43,44,45,46,54]:

- heating of the metal surface;
- melting of the metal surface;
- spallation of surface layers (rupture of material when the fluence is above the ablation threshold);
- phase explosion (explosive boiling of the surface with the generation of vapor and plasma).

**Figure 4:**
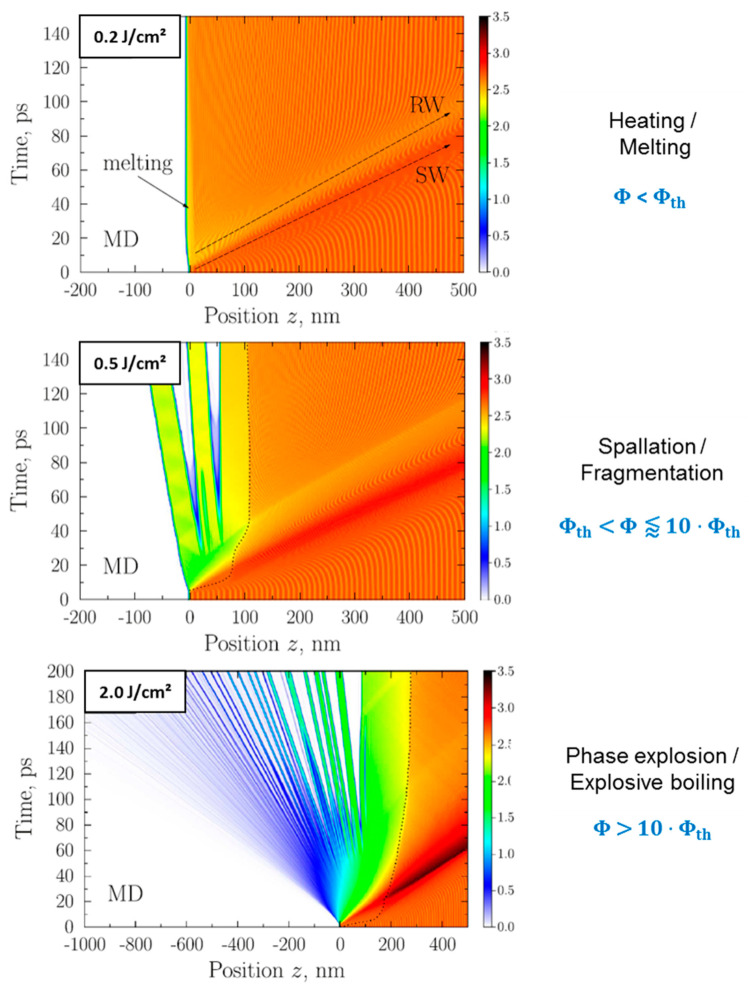
Results from molecular dynamics (MD) simulations for single pulse irradiation of aluminum. The *x*-axis corresponds to spatial coordinates with z = 0 nm being the initial surface of the material. The *y*-axis corresponds to the simulation time with t = 5 ps being the maximum of the laser pulse. The color of the heat maps corresponds to the density of aluminum. With increasing fluence (from **top** to **bottom**), the material passes different regimes, ranging from melting of the surface (no ablation) to phase explosion with an excessive development of gaseous and ionized matter. In the spallation regime (**center**), ablation is characterized by a fragmentation of the material, leading to high surface qualities and well-defined ablation depths during laser processing. Adapted from [46], copyright 2015, with permission from Elsevier.

A microscopic view of the occurring processes for ablation of aluminum is given in Figure 4 [46]. When a laser pulse hits the target with a fluence below the ablation threshold, the material is heated and melting of the surface may also take place. Furthermore, two sorts of pressure waves propagate into the material from the surface. A shockwave (SW) leading to a compression of material (higher density, darker orange color) is followed by a rarefaction wave (RW), leading to a dilution of material (lower density, lighter orange color). When the fluence is increased above the ablation threshold, the intensity of both pressure waves is increased and, as a result of the lower density of the rarefaction wave, a fragmentation of surface layers takes place (spallation regime). The travel velocity of such dense layers evolving from the surface for metals lies in the range of several 100 m/s [45,46,54,55,56,57]. If the fluence is increased above values of approximately 10 times the ablation threshold, not only spallation takes place but the upper surface layers (in this representation on the left) disintegrate and show a transition into the gaseous phase. Particles and clusters in the gaseous phase then travel principally with the speed of sound (of the initial bulk material), which lies in the range of several km/s [45,46,54,55,56,57].

**Figure 5:**
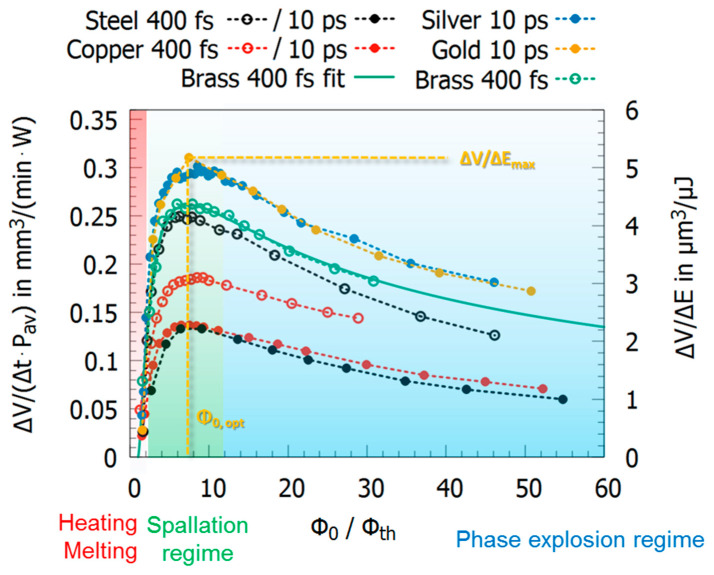
Energy specific volume for single pulse laser ablation of different metals processed in the near infrared. The fluence along the *x*-axis is normalized to the ablation threshold since different metals have different ablation thresholds. The corresponding values are given in Table 2. Original data taken from [59,60].

Although not displayed here, atoms in the gaseous phase also become ionized for higher fluences, and some regions reach temperatures of several 1000 K or more [45,46,54,55,56,57]. If successive pulses have inter-pulse delays of several tens of microseconds or more (corresponding to repetition rates of $f_{rep}=$ 100 kHz or less), a subsequent pulse does not significantly interact with the ablation plume of its preceding pulse [58]. For laser material processing, the different ablation regimes result in differently efficient micromachining processes. One quantity for the description of an efficient ablation process is the energy specific volume ΔV/ΔE. For Gaussian laser beams it holds [14]:

(4) $$\frac{\Delta V}{\Delta E}=\frac{1}{2}\cdot \frac{\delta }{\phi_{o}}\cdot \ln^{2}\left(\frac{\phi_{0}}{\phi_{th}}\right)$$

where δ corresponds to the effective penetration depth of energy, $\phi_{0}$ is the incident peak fluence, and $\phi_{th}$ is the ablation threshold. Experimental data and, for the sake of clarity, only one fit function (brass, 400 fs, cyan curve) is given in Figure 5. The values were obtained in laser milling experiments by ablating cavities (cf. Figure 1c), measuring the ablated volume, and relating it to the incident total laser energy. The values follow the trend of Formula (4), having a maximum that can be extracted from the data. The corresponding values of δ, $\phi_{th}$, and the fluence $\phi_{0,opt}$ where the maximum value was achieved and the maximum energy specific volume $\Delta V/\Delta E_{\;max}$ (maximum values in Figure 5) as well as the experimental conditions such as wavelength and pulse duration are summarized in Table 2.

For single pulse operation, this formula resembles well the experimental findings. The different metals show different maximum energy specific volumes and ablation thresholds, but the general trend is the same. Principally, such curves are found for metals when processed with pulses with a pulse duration between a few 100 fs and several ns [60,61].

Independent of the metals investigated, the ablation process when using ultra-short pulses is most efficient in the spallation regime, since the greatest amount of deposited energy is used for ablation. For lower fluences, only heating of the material takes place, whereas for much higher fluences the amount of energy being used for heating and ionization increases [62].

Beside the ablated volume per energy, the ablated volume per time (a measure for the productivity) is a very important parameter for micromachining processes such as milling or drilling. It increases for higher fluences, while the ablation process itself becomes less energetically efficient (∆V/∆E decreases). At the same time, low fluences close to the ablation threshold (or several times the ablation threshold) correlate with low surface roughness and less spatter occurrence [14,63,64]. For industrial micromachining processes, it is often important to maintain a smooth surface while keeping the ablated volume per time high. Since the available laser pulse energy and average power of industrial laser systems have been significantly increasing within the last decade, the available energy per pulse is often much higher than the energy needed for ablation. Among other strategies such as defocusing of the processing beam to lower the fluence while keeping the productivity at a high level [18,65], the concept of laser burst pulses was introduced, allowing the principle use of the totally available laser energy by splitting it into several pulses with lower energy each [66,67]. The resulting limitations of this concept will be further discussed in the following sections.

Beside the ablated volume per energy and time, the residual energy is another important quantity influencing micromachining processes, mainly in terms of surface quality, e.g., surface roughness. If a sufficiently high amount of energy is coupled into the machined sample as heat, the surface structure and physical mechanism of material removal may change. For example, a temporary molten surface can lead to higher roughness and spatter production, decreasing the surface quality. During ablation with an ultra-short pulse, a certain amount of energy remains as heat in the bulk material. Depending on the irradiated metal and the used fluence, the amount of residual energy per pulse lies between 10% and 50% [16,17,68,69,70,71,72,73,74,75,76]. If high repetition rates are used, the heat accumulates and can affect the surface quality, the drilling time, and the amount of occurring melt [14,73,77,78,79,80,81,82,83,84]. Additionally, the amount of residual energy can increase when the geometry is changed, e.g., in hole drilling [68,70,72,74], or when surface structures like laser-induced periodic surface structures (LIPSS) or bumps evolve [69,84].

### 2.2. Double Pulses: Pulse-to-Pulse Interaction

The first investigations of laser treatment of metals with ultra-short double pulses go back to around the turn of the 21st century, when investigations about the enhancement of spectroscopic signals—today also often known as laser-induced breakdown spectroscopy (LIBS)—with ultra-short double pulses were performed [7,85,86,87]. While it was shown that signal enhancement was possible, the depths of the ablation craters formed showed a special behavior, i.e., the crater depths could only be as deep as using a single pulse or even less deep, which is not intuitive in the first place, since twice as much energy was used to create these craters. As will be summarized in the following, both effect signal enhancement from the irradiated plume as well as the development of less deep craters are mutually dependent. Many experimental [7,8,14,16,19,57,58,59,66,88,89,90,91,92,93,94,95,96,97,98,99,100,101,102,103,104,105,106,107,108,109,110,111,112,113,114] and numerical [9,10,12,54,93,115,116,117,118,119] studies have been performed since the first observations about metal ablation with ultra-short double pulses were published. The following paragraphs summarize the findings to provide an overview of the effects involved in ultra-short double pulse laser ablation.

**Figure 6:**
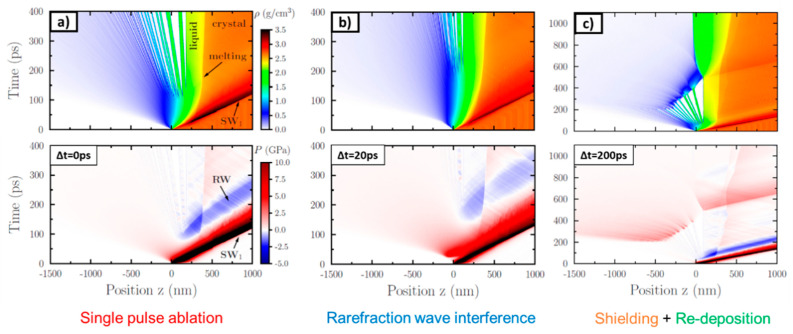
(**a**–**c**) Results from MD simulations showing the density (**top**) and pressure (**bottom**) distributions in space and time for different inter-pulse delays of double pulses. Material: aluminum, pulse duration 100 fs, wavelength 800 nm. Adapted with permission from Ref. [10]. Copyright (2015) by the American Physical Society.

**Figure 7:**
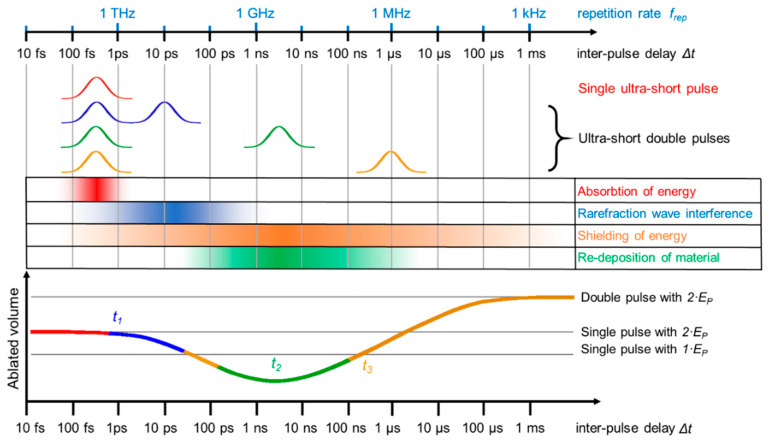
Interaction mechanisms of metals and ultra-short single and double pulses for different inter-pulse delays.

When a double pulse with short inter-pulse delay times in the range of the electron–phonon coupling time is used, the ablated volume of such a double pulse equals the volume ablated by a single pulse with doubled pulse energy. With increasing inter-pulse delay, several physical effects can occur. For delays in the range of 10 ps to a few 100 ps, rarefaction wave interference takes place. As shown in Figure 4 (center), the ablation process of single ultra-short pulses is characterized by the occurrence of a shock wave and a following rarefaction wave. The intensity of the rarefaction wave is a critical parameter of a successful ablation event. If a rarefaction wave is too weak, no ablation occurs (cf. Figure 4 (top)). For double pulse ablation, weakening of such rarefaction waves is an important process. Exemplarily, calculated density and pressure distributions for different regimes of double pulse laser ablation are given in Figure 6 [10]. For the pressure distributions given in Figure 6, red regions denote regions with compressive pressures, whereas blue regions denote regions of tension. Figure 6a shows the result of double pulse irradiation with an inter-pulse delay of Δt= 0 ps (corresponding to a single pulse of double pulse energy). An intense shockwave is followed by an intense rarefaction wave. From the regions of highest tension (blue), ablation is initiated. If the inter-pulse delay is increased to around Δt= 20 ps, the first shockwave is weaker, since this wave is only initiated by the first pulse. The following rarefaction wave is less pronounced because the second pulse increases the pressure and, hence, lowers tension. As a result, less material is removed and the ablation depth is decreased (cf. Figure 6b). This effect of “rarefaction interference” is the reason for a lowering of the ablation depth for metals if the pulses are separated by several tens of picoseconds (blue region in Figure 7) [10,120]. If the inter-pulse delay is increased to Δt= 50 ps, the second pulse interacts with material that has already left the initial metal surface and no physical connection for the exchange of pressure remains. Instead, the second pulse is shielded by the ablation cloud induced by the first pulse [9,10,54,57,118,119,120]. The ablation cloud evolving from the surface after the first pulse is heated and partially disintegrates, leading to an extreme increase in temperature and plasma formation.

The latter is an important effect beneficial for LIBS measurements and allows a better characterization of material due to an increased plasma temperature and, hence, a more pronounced spectrum (cf. Section 3.5.2). Depending on the fluence, not only the second pulse is shielded (and is not contributing to further material removal) but a re-deposition of the material ablated by the first pulse can take place (cf. Figure 6c). As a result, the ablated volume of a double pulse can be lower than the volume ablated only by the first pulse (valley of death) [57,75,95,103,109,111,121,122,123]. This effect is material- and fluence-dependent. For example, the valley of death for stainless steel ranges between 100 and 200 ps at a fluence of 0.5 J/cm^2^ [75] and shows a minimum at around 50 ps for 5 J/cm^2^ [121]. Copper shows a valley of death for inter-pulse delays between 100 ps and 2 ns at fluences of 0.69 J/cm^2^ [57] and 5 J/cm^2^ [121]. Aluminum shows a valley of death between 100 and 300 ps for an incident fluence of 5 J/cm^2^ [121]. Titanium and platinum show valleys of death between 100 and 500 ps for incident fluences close to the ablation threshold [123]. For steel, a broader study with different time delays and fluences was also performed, and it was reported that the highest suppression of ablation takes place for steel at around a 1 ns time delay. The highest suppression ratio was close to 10, so only 10% of the volume ablated by a single pulse was finally removed using a double pulse (parameters: wavelength 1028 nm, fluence approx. 2.3 J/cm^2^) [103]. If the inter-pulse delay is increased to several tens of nanoseconds, the shielding and re-deposition becomes less pronounced, since the ablation cloud formed after the first pulse dilutes with increasing time delay. For inter-pulse delays of Δt> 10 µs, no significant interaction of the second pulse and the plume induced by the first pulse occurs anymore [58]. The discussed principal physical effects are summarized in Figure 7. Of course, the exact time delays, fluences, and pulse durations when these effects occur are material-dependent. The most significant differences between the materials are the time delay $t_{1}$, when the decrease of volume starts (blue region in Figure 7), the time delay $t_{2}$, when the minimum of ablated volume is reached, and the time delay $t_{3}$, when the re-deposition is not taking place anymore and the volume ablated by a single pulse is reached again (transition from the green to the yellow region in Figure 7).

For short delay times in the range of the electron–phonon coupling time (red color), the volume ablated by a double pulse equals the volume ablated by a single pulse with doubled pulse energy and the ablation process the same as for a single pulse, cf. Figure 4 and Figure 6a. For delays in the range of 10 ps to a few 100 ps, rarefaction wave interference takes place, cf. Figure 6b. For inter-pulse delays between a few 100 ps and several 100 ns, a shielding of the second pulse by the ablated material of the first pulse takes place. For inter-pulse delays of a few nanoseconds, re-deposition of material ablated by the first pulse is another effect that can occur, cf. Figure 6c. The shielding effect diminishes for longer inter-pulse delays of several tens of nanoseconds due to the dilution of the plume initially induced by the first pulse. For delays of several tens of microseconds and longer, the second pulse does not interact with the plume, and the volume ablated by a double pulse is twice the volume ablated by a single pulse.

Although it is still the subject of current research, it seems that theses characteristic delay times are dependent on the electron–phonon coupling time. If the second pulse is reaching the target before the transfer of energy between the electronic and ionic system is finished, the material mainly reacts similar to an irradiation with a single pulse of double energy. For example, steel has a much shorter electron–phonon coupling time than copper (approx. 50 times lower), and the decrease in ablated volume starts from shorter time delays ($t_{1}\;\approx$ 1 ps for steel, $t_{1}\;\approx$ 10 ps for copper) [75,103]. For all metals investigated in the literature and as discussed before, t_2_ lies between time delays of several 100 ps and several nanoseconds. For steel and copper, the difference is significant, e.g., for the fluences: 0.5 J/cm^2^ for steel and 0.69 J/cm^2^ for copper in the infrared, and $t_{2}$ for steel amounts to ~200 ps, whereas for copper $t_{2}$ amounts to ~2 ns [75]. Additionally, the delay time $t_{3}$, when the volume ablated by the double pulse again is the same as for a single pulse, differs for both metals in this fluence regime. For steel, $t_{3}$ amounts to approximately 1–3 ns [75,103,122], whereas for copper $t_{3}>$ 10 ns [75,122]. For aluminum, it was also reported that $t_{3}>$ 10 ns [122].

### 2.3. Triple Pulses with Intra-Burst Delays in the Range of Tens of Nanoseconds

**Figure 8:**
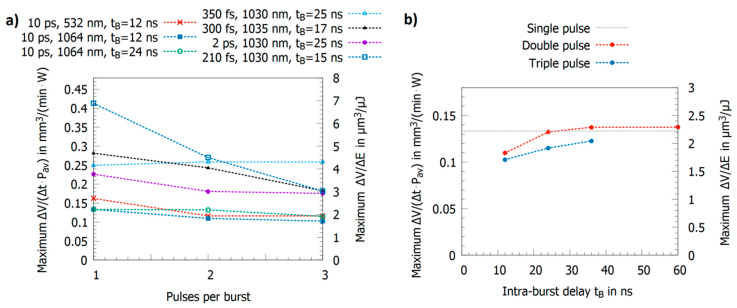
Maximum measured energy specific volumes for single, double, and triple pulses for steel 1.4301 (AISI 304) for (**a**) different pulse durations, wavelengths, and intra-burst delays and (**b**) as a function of the intra-burst delay for double and triple burst pulses with a 10 ps pulse duration and a 1064 nm wavelength. Data taken from [14,19,23,24,102,107].

As described in the previous section, the characteristic delay times $t_{1}$, $t_{2},$ and $t_{3}$ strongly depend on the material, especially on the electron–phonon coupling time, which also has a dominating influence onto the removal rates when a third pulse is added to the double pulse. If $t_{1}$ and $t_{2}$ are short compared to the intra-burst delay and $t_{3}$ shows a value in the range of this delay, the energy specific volume obtained for a triple pulse only slightly differs from the one of single pulses, e.g., steel 1.4301 (AISI 304) shows such short delay times and was intensively investigated. Different reported maximum energy specific volumes for single, double, and triple pulses are summarized in Figure 8a [14,19,23,24,102,107]. For pulses with a pulse duration of 10 ps, an intra-burst delay of 12 ns, and wavelengths of 1064 nm and 532 nm, the maximum energy specific volume slightly decreased for a triple pulse compared to a single pulse [14,102,107]. An almost identical behavior was observed for a 2 ps pulse duration but with higher ablated volumes due to the usage of shorter pulses [19]. Similar results with even higher energy specific volumes have been reported for 300 fs and 350 fs pulse duration in the near infrared (NIR) [23,60]. In contrast to the rest of the literature, in [24], a significant drop by a factor of two of the maximum energy specific volume for steel (from about 0.4 mm^3^/min/W for single pulses down to less than 0.2 mm^3^/min/W, Figure 8a, blue empty squares) for a triple pulse burst was reported for a 210 fs pulse duration for a wavelength of 1030 nm. Here, in contrast to most other reported experiments by different authors, the fluence was varied by defocusing. This leads to different spot sizes at the fluences where the maximum energy specific volume was detected for the three situations. It was found for single pulse operation that there is a dependence of the threshold fluence, the energy penetration depth, and the maximum energy specific volume for steel 1.4301 (AISI 304) and copper when using different spot sizes [124]. This could serve as a possible explanation for the atypical behavior reported in [24] in contrast to all other studies in this field. The influence of the spot size on laser milling in general, but especially for bursts, still needs to be clarified by further studies.

Figure 8b shows the maximum energy specific volume for steel 1.4301 (AISI 304) using pulses with a pulse duration of 10 ps at a wavelength of 1064 nm and an intra-burst delay increasing from 12 ns to 36 ns for triple pulses and 60 ns for double pulses [14]. For the shortest intra-burst delay of 12 ns, the maximum volumes are smaller than the value for single pulse operation. For double pulses with an intra-burst delay of 24 ns and more, the same maximum value as for single pulse operation is reached (deviations are in the range of the measurement error). For triple pulses, the maximum energy specific volume also increases with increasing intra-burst delay but does not yet reach the value of single pulse operation with a 36 ns pulse delay.

**Figure 9:**
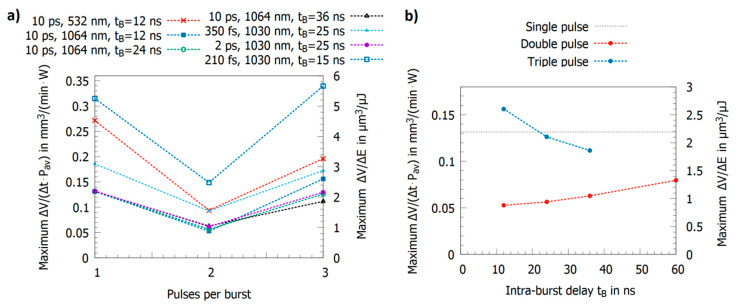
Maximum measured energy specific volumes for single, double, and triple pulses for (**a**) different pulse durations, wavelengths, and intra-burst delays for copper and (**b**) as a function of the intra-burst delay for double and triple burst pulses on copper with 10 ps pulse duration and 1064 nm wavelength. Data taken from [14,19,23,24,102,107].

The situation completely changes for copper (having longer delay times $t_{1}$, $t_{2},$ and $t_{3}$) as shown in Figure 9a,b. For double pulses, the aforementioned valley of death with a reduction in the maximum energy specific volume to less than 50% of the value for single pulses due to shielding effects is observed [14,24,59,60,102]. On the other hand, for triple pulse processing, the value strongly increases again and may even exceed the value for single pulses [14,24,59,60,102]. As can be seen in Figure 9a, the maximum energy specific volumes are functions of the pulse duration (an increase for shorter pulse durations is observed) and wavelength (for copper, green radiation is beneficial and results in higher maximum energy specific volumes, which is mainly due to higher absorptance [16]). For triple pulses, in the first place, it seems that after the described re-deposition effect (cf. Section 2.2.) induced by the second pulse, the third pulse should simply contribute again to the ablated volume. However, assuming only a complete shielding and no re-deposition of the second pulse (no contribution to the ablated volume) and a full contribution of the third pulse to the ablated volume, the maximum energy specific volume should not exceed two-thirds of the value of single pulses. This is not the case. Indeed, for triple pulse processing, the maximum energy specific volume is the same or even higher than for single pulse processing.

The contribution of the second pulse was further investigated in [102,125], where the energy of the second pulse was varied compared to the first and third pulse, i.e., the relative energies were chosen to be (1,η,1). The pulses had a pulse duration of 10 ps, a wavelength of 1064 nm, and an intra-burst delay of 12 ns. For the situation (1, 0.25, 1), the energy specific volume does not differ from the one of a double pulse with an intra-burst delay of 24 ns. When the energy of the second pulse is then raised to (1, 0.5, 1), the maximum energy specific volume is almost doubled and even exceeds that for single pulses for the (1, 0.75, 1) and (1, 1, 1) situations. This shows that a minimum energy for the second pulse is demanded to start its clearing effect and that it saturates at a maximum energy. However, the high maximum energy specific volume for a triple pulse cannot be explained only by this effect.

Therefore, it was stated that additional effects influence the ablation process in case of triple burst pulses on copper to allow an enhanced ablation. In [16,17], results from calorimetric measurements with 10 ps pulse duration were obtained from two different calorimeter set-ups. For copper, it is shown that the residual energy coefficient, resembling the part of the applied laser energy remaining and heating up in the material only slightly varies between single, double, and triple pulses processing. For both measurement setups, the residual energy coefficient was measured to be slightly higher (an increase in the range of 2%) for the double pulse, but the difference was in the order of the measurement error. However, the absorptance of the machined surface changes significantly when using lasers in the NIR. The corresponding absorptance increased from 1.5% of the initial surface (high reflectivity) to 15.7%, 17.9%, and 26.8% for single, double, and triple pulses, respectively. This increase was obtained from the first machined layer and rests almost unchanged when the number of layers was increased to 48. For a triple pulse, the first pulse in the burst sequence could be absorbed much more strongly compared to single pulses, since 26.8% of incident energy was absorbed instead of 15.7% [16]. This could explain the further increase in the maximum energy specific volume for triple pulses on copper. This assumption is further supported by the results obtained for a wavelength of 532 nm, where the absorptance of the machined surface again increases from 35.7% for single pulses to 49.5% and 58.5% for double and triple pulses, respectively. This increase was lower than for 1064 nm, which is in accordance with the smaller increase in the energy specific volume for 532 nm and the triple pulse burst shown in Figure 9a. Hence, the absorptance seems to play a crucial role, and, due to significantly increased absorptance after the second pulse, the energy specific volume of copper might be increased to or even above the value of the single pulse operation. It should be kept in mind that these absorptances were measured on a cold surface ca. 10 mm out of the focal plane and therefore with a spot diameter between 400 µm and 700 µm, whereas, during machining, the surface is hot, and the spot diameter is in the range of 25 µm to 40 µm. Furthermore, the absorptance of a surface can also vary during irradiation with an ultra-short pulse, as shown in [126]. How this behavior affects burst pulse processing is unknown today and still a subject to further investigation.

In Figure 9b, the maximum energy specific volumes for double and triple pulses for pulses with a pulse duration of 10 ps at a wavelength of 1064 nm are shown as a function of the intra-burst delay $t_{B}$ [17]. Due to the longer delay times $t_{1}$, $t_{2},$ and $t_{3}$ of copper, the values seem to increase for intra-burst delays exceeding 60 ns in contrast to steel, where the single pulse value is already obtained for an intra-burst delay of 24 ns, as illustrated in Figure 8b. However, in contrast to steel, the maximum energy specific volume decreases for triple pulses when the intra-burst delay is increasing (cf. Figure 9b). This is a surprising behavior as the shielding effect of the second pulse is definitively reduced. A possible explanation is that since less material is redeposited, more material remains above the workpiece in a less dense ablation cloud, leading then to a shielding of the third pulse. However, this is only a hypothesis and further investigations are needed to clarify the physical processes involved during triple pulse ablation.

**Figure 10:**
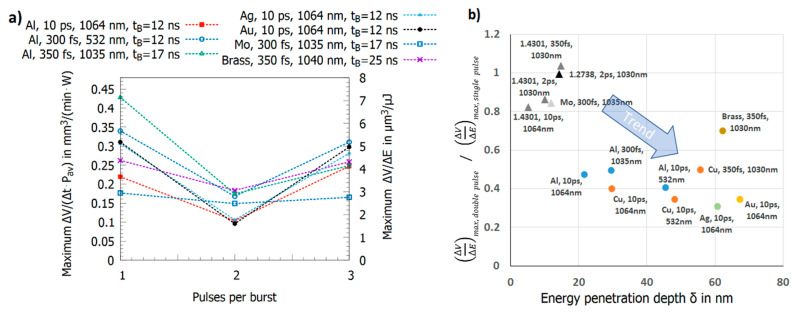
Maximum energy specific volumes for single, double, and triple pulses for (**a**) brass, aluminum, silver, gold, and molybdenum and (**b**) the ratio of the maximum energy specific volume of a double burst pulse and single pulses as a function of the corresponding energy penetration depth for different metals. The triangles denote metals where the energy specific volume of a double and triple pulse burst does not show a shielding effect for the double pulse burst followed by an increase in the energy specific volume for the third pulse within a triple pulse. Data taken from [19,23,24,60,128,129].

Figure 10a shows the maximum energy specific volumes for brass [60], aluminum [23,107], silver [59], gold [59], and molybdenum [23]. Except for molybdenum, a strong shielding effect for the double pulse burst followed by a significant increase in the maximum energy specific volume for the triple burst pulse is observed. Following Formula (4), the energy specific volume for a Gaussian beam shows a maximum value at the optimum peak fluence $\phi_{0,opt}=e^{2}\cdot \phi_{th}$ [61]. Introducing this optimum peak fluence into the expression for the energy specific volume (Formula (4)) leads to the ablated volume per pulse at the optimum fluence, which does not depend on the threshold fluence but directly scales with the energy penetration depth δ [65,127], i.e., the higher the energy penetration depth, the higher the volume ablated per pulse. The value of δ can be obtained by a least square fit of the formula to the data for single pulses reported in [14,23,59,60,107,128] following Formula (4). A larger ablated volume should lead to a stronger shielding effect for double pulses and therefore the ratio

(5) $${\left(\frac{\Delta V}{\Delta E}\right)}_{max,double\;pulse}/{\left(\frac{\Delta V}{\Delta E}\right)}_{max,single\;pulse}$$

should drop for higher energy penetration depths. This is confirmed in Figure 10b where this ratio is plotted as a function of the energy penetration depth δ. The circles denote situations where a clear shielding for the double burst pulse followed by a significant increase of the energy specific volume for the triple burst pulse is observed, whereas for the triangles, the maximum energy specific volumes for the three situations only slightly differ. The figure indicates a trend of ratios near 1 for short energy penetration depths in the range of 10 nm towards values below 0.5 for energy penetration depths of 40 nm and higher. Hence, the removed volume per single pulse could also be a factor describing the ablation behavior for double and triple pulses. Again, further investigations are needed to gain a clearer picture and to be able to distinguish between the influence of parameters such as the spot size, the intra-burst delay, and the energy penetration depth.

### 2.4. Multi-Pulse Bursts with Intra-Burst Repetition Rates in the MHz Range

**Figure 11:**
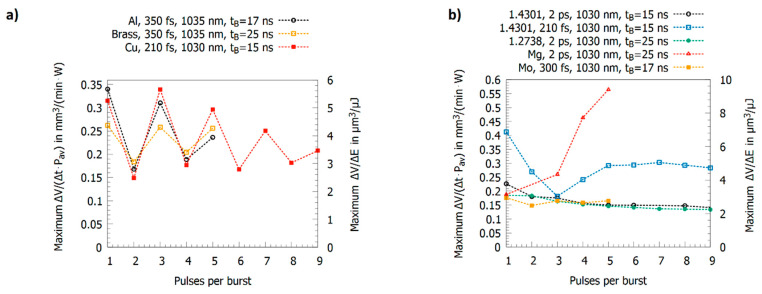
Maximum energy specific volumes as a function of the number of pulses per burst for (**a**) copper, brass, and aluminum as well as for (**b**) stainless steel, molybdenum, and magnesium alloy. Data extracted from [19,23,24,60,128,129].

The results for multi-pulse bursts for copper, brass, and aluminum are summarized in Figure 11a [19,23,24,60]. Copper, brass, and aluminum show an alternating behavior of the maximum energy specific volumes with high values for odd numbers of pulses in the burst and low values for even numbers of pulses. This can be explained as follows: the second pulse is fully or partially shielded but its energy clears the ablation cloud such that the third pulse can be absorbed by the surface. The third pulse again generates an ablation cloud, and the fourth pulse is therefore shielded again. The fourth pulse again clears the plume of the third pulse so that the fifth pulse can again contribute to the ablation process etc. For copper and a 210 fs pulse duration [24], aluminum and a 300 fs pulse duration [23], and brass and a 350 fs pulse duration [60], this oscillation seems to be damped.

In contrast to this oscillating behavior, this alternating behavior is not observed for steel 1.4301 (AISI 304) [19,24], steel 1.2738 [130], molybdenum [23], and magnesium (cf. Figure 11b). For steel and molybdenum, the maximum energy specific volume shows a tendency to slightly lower values when the number of pulses per burst is increased. Steel 1.4301 (AISI 304) machined with 210 fs pulses [24] shows an atypical behavior up to 4 pulses per burst, but these results were obtained by defocusing to vary the peak fluence. Hence, different effects cannot be distinguished, as has been explained before. For magnesium alloy [129], the threshold fluence and the energy penetration depth amounts to 0.06 J/cm^2^ and 6.5 nm, respectively. Therefore, a similar behavior as for steel 1.4301 (AISI 304) could be expected, but, as can be seen, the maximum energy specific volume increases by about a factor of three when the number of pulses per burst is raised from one to five. This is in contrast to the other metals and is similar to the behavior of silicon [17], where the maximum energy specific volume also increases with the number of pulses per burst. For silicon, this increase of energy specific volume is mainly due to changes in absorptance due to multi-photon absorption and stable liquid surface layers (having a higher absorptance) during processing [17].

Recently, the behavior of stainless steel was investigated for pulses at a wavelength of 1030 nm and with pulse durations of 270 fs, 1 ps, and 10 ps for single pulses for up to 9 pulses per burst with an intra-burst delay of 15.4 ns. The results show that the energy specific volumes for peak fluences of 0.5 J/cm^2^, 1.5 J/cm^2^, and 2.5 J/cm^2^ mainly depend on the pulse duration, but follow identical trends when the number of pulses per burst is increased. Depending on the pulse duration and the peak fluence, regimes where a melt film is formed leading to smooth surfaces can be identified [131]. Similar smoothing effects were already reported for steel 1.4301 (AISI 304) in [14,60] and for cobalt and titanium alloys in [78,132]. Neither for copper nor for brass could a smoothing effect be observed, and the surfaces only start to oxidize when they are machined with pulse bursts [14,60].

In contrast to double pulses, the theory behind triple pulse ablation still shows an ambiguous picture. To allow for a better understanding of the physical mechanisms taking place during processing with triple pulses, further investigations with varying intra-burst delays need to be performed.

### 2.5. Multi-Pulse Bursts with Intra-Burst Repetition Rates in the GHz Range

In 2016, a new type of laser emitting ultra-short burst pulses with intra-burst repetition rates in the GHz range, and, hence, with intra-burst delays of several nanoseconds and down to a few 100 ps, gained attraction for material processing due to a report showing that the efficiency of the ablation process was significantly improved for a variety of materials [15]. However, the reported physical mechanism influencing the ablation process, the “ablation cooling effect,” appears highly questionable and has been partially disproven for metal processing. Since it can be mainly attributed to heat accumulation [133,134,135], the publication was the starting point for materials processing with a completely new class of laser systems emitting pulse bursts with intra-burst delays of several 100 ps or at intra-burst repetition rates of several GHz, which have since then been built and studied by various research groups. In general, it can be concluded that drilling processes are always more efficient than milling processes in this processing regime [135,136], which was explained by different melt flows in those two regimes [133]. Furthermore, the increase in energy specific volume is highly dependent on the time span of the used burst train and increases with longer time spans [22,136,137,138,139]. Moreover, the used inter-burst delay plays a crucial role. For short time delays (corresponding to higher repetition rates) between the burst trains, heat accumulation is also more pronounced and contributes to an increase in energy specific volume [112].

It has been reported by several groups that both the ablated volume per time ΔV/Δt and the energy specific volume ΔV/ΔE can be increased by several factors when processing material with ultra-short burst pulses with intra-burst repetition rates in the GHz regime compared to single pulse operation. For the milling of copper, it was reported that the maximum energy specific volume $\Delta V/\Delta E_{max}$ can increase by a factor of three [135], whereas for steel it can increase by a factor of two when using GHz burst pulses instead of single pulses [112,135,137]. For the drilling of copper, it was reported that the maximum energy specific volume $\Delta V/\Delta E_{max}$ can increase by a factor of 3.5; for steel, it can increase by a factor of 4.6; and, for aluminum, it can increase by a factor of 5.8 when using GHz burst pulses instead of single pulses [136]. All these effects diminish and are less pronounced when during experimental design the spot diameters on the samples investigated are also changed for optimization [24].

**Figure 12:**
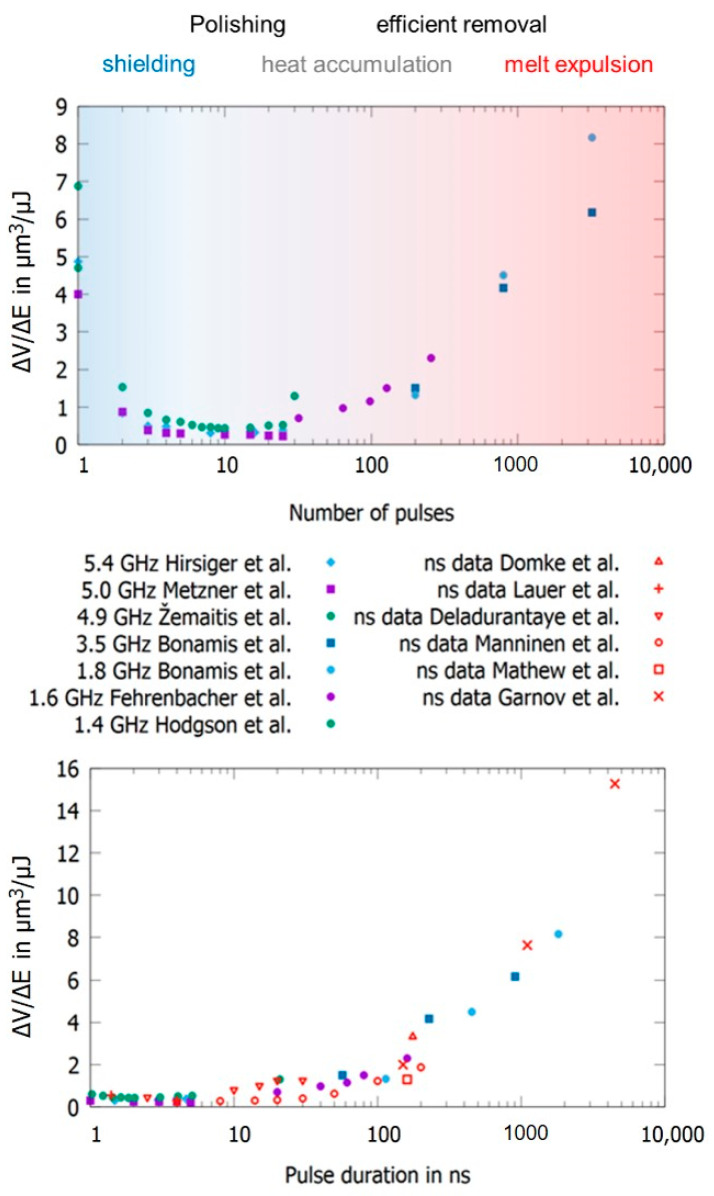
Maximum achievable energy specific volumes as a function of applied laser pulses within GHz bursts (**top**) and maximum energy specific volumes as a function of the duration of GHz bursts and durations of ns and µs pulses (**bottom**). Data from laser milling of stainless steel, data extracted from [21,23,24,64,98,124,135,141,142,143,144,145,146,147].

To be able to account for different study designs, a comprehensive overview of data from different authors is given in Figure 12 (top) for laser milling using GHz laser sources. When using double pulses, the previously discussed shielding effect is dominant, and the maximum achievable energy specific volume drops by a factor of five for intra-burst delays in the range of several 100 ps. Furthermore, with the increasing number of burst pulses the decrease in maximum achievable energy specific volume drops even further and is suppressed by approximately 93% for steel when using 5 to 25 pulses in a pulse burst [23,24]. From around 30 pulses per burst the energy specific volume increases again, and, for several hundred or thousands of pulses per burst, it even increases above the value for single pulse ablation for both copper and steel. For a small number of pulses within a burst, a region of higher density in the interaction zone of the laser and the target develops, and the shielding effect is even more pronounced [134]. For a moderate number of pulses (several tens to hundred pulses), the ablation process is dominated by heat accumulation, allowing material to be removed in a gaseous state even for pulses with a fluence far below the ablation threshold (which is the case because the totally available laser pulse energy is principally split between many pulses within the burst) [133,134,135,140]. For a high numbers of pulses (several hundred to many thousand pulses) within a burst, the ablation process can be compared to ablation with pulses of nanosecond duration, resulting in higher energy specific volumes as well as a lowering of the surface quality due to melt expulsion, which indeed is the case for GHz laser ablation [64,135,136]. This simple but fair comparison is given in Figure 12 (bottom), where the maximum achievable energy specific volumes as a function of the time span of burst packages are compared to the energy specific volumes of ns and µs laser processes. It can be seen that the maximum energy specific volumes of GHz laser processes fully follow the ones of ns and µs lasers in the same time domain. This is not only the case for steel but also for copper (not displayed here). Therefore, it can be concluded that GHz processing is comparable to ns laser processing for pulses of the same time span for metals. Of course, more detailed studies need to be performed to gain a full view on this topic. Finally, it can be seen from the reviewed and summarized studies that the surface roughness shows a minimum for a low number of burst pulses (corresponding to short burst time spans), which can be significantly lower compared to single pulses [21,23,24,64,124,135,141,142,143,144]. For longer time spans and a higher number of pulses, heat accumulation highly dominates laser processing, leading to higher energy specific volumes as well as significantly higher roughness, which is also the case for longer pulses in the ns and µs regime due to the emerging occurrence of melting.

Beside materials processing with ultra-short burst pulses with intra-burst repetition rates in the MHz and GHz range, processing with THz intra-burst repetition rates (corresponding to delay times between subsequent pulses of several ps) is also a subject of investigation [20,148,149,150,151,152]. However, in this regime, matter reacts to excitation with subsequent pulses similar like to excitation with longer pulses in the same time domain. Beside a dependence of the incubation effect (a weakening of material) on the burst spacing [149,153], the material answer is similar when using multiple subsequent ultra-short pulses over a certain time span and when using a single pulse with a pulse duration of the same time span, e.g., a pulse burst with 10 pulses that are applied over a time span of 10 ps shows similar effects to a pulse with a 10 ps pulse duration. Hence, a differentiation of physical effects on these time scales is challenging. From the material processing point of view, the use of burst pulses with THz intra-burst repetition rates is less important but has found its way into the generation of particle beams and X-rays (cf. Section 3.5.3).

## 3. Applications Using Burst Pulses

In the following subsections, applications involving pulse burst processing of metals are summarized and discussed. The focus mainly lies on micromachining applications, while applications from other fields making use of pulse bursts are also summarized in the last subsection.

### 3.1. Punching and Drilling

Drilling with ultra-short pulsed laser radiation has been a widely used production technique for making precise holes in various materials for a long time. A large number of laser pulses and a sufficiently high pulse energy is typically required to produce blind and through holes with high aspect ratios of 1:10 or more. The reason lies in the nature of the drilling process, where, in general, the irradiated area increases with drilling depth and pulse number, and, hence, the fluence within the bore holes decreases over time [74,154,155]. Principally, this requirement contradicts the splitting of pulse energy during burst processing to allow for an improvement in laser drilling processes in terms of drilling time and, hence, efficiency. The same holds for an increase in the pulse repetition rate, since shielding effects can be even more pronounced due to lateral confinement of the ablation cloud. Laser drilling involves interaction of the radiation with ablation products from previous laser pulses. Shorter time spans between successive laser pulses intensify this effect [57,61].

There have been comparatively few studies on the influence of bursts in laser drilling with ultra-short pulses. A particularly early investigation into percussion drilling with MHz bursts of aluminum showed advantages in terms of drilling time [156]. However, these advantages must be seen in the context of the repetition rates in the kHz range that were common at the time. The main advantage was therefore the higher number of pulses per time achieved by the burst, resulting in more ablated volume per time.

Interactions of burst pulses with the ablation products within bore holes were investigated diagnostically, whereas drilling glass was investigated by time-resolved imaging [157]. As the investigated material was not a metal, the observed effects are presumably very similar. Although there appeared to exist shielding effects of radiation within the bore hole, the holes obtained showed a more uniform geometry with smoother walls. Similar observations were made when drilling silicon [158], where processing with MHz burst resulted in fewer unwanted side channels and overall higher reproducibility of the hole shape. This is highly due to re-deposited material, leading to smoother surfaces.

In [159], the drilling of copper and aluminum was studied with double pulses, varying the inter-pulse delay from −90 ps to 90 ps. Here, the observed reduction in drilling speed in the case of using double pulses was attributed to shielding as well. This is clearly in contrast to the conclusions provided in [15], where much higher removal rates were reported for machining copper and silicon with GHz bursts. In [133], an attempt was made to reproduce these advantages, but significant melting was observed; so, as discussed before, GHz processing seems to have similarities with the use of nanosecond pulse durations. It was shown that the maximum energy specific volumes achievable when using a burst with 200 pulses at an intra-burst repetition rate of 1.76 GHz are almost identical when processing with a laser at a pulse duration of 100 ns for percussion drilling of copper, aluminum, and stainless steel [64,135].

A study about laser drilling of invar with ultra-short MHz and GHz bursts has shown an ambiguous picture. Depending on the sample thickness and intra-burst delay, slightly shorter drilling durations or even longer drilling durations were reported compared to drilling without bursts [137].

Most studies on laser drilling with ultra-short burst pulses show a rather minor influence on the drilling speed but an increased melt formation. With regard to the question of how far the special properties of bursts can be used to advantage in laser drilling, there is still a considerable need for research.

### 3.2. Scribing and Cutting

In several works, line scribing using bursts have been performed [22,133,135,151,160] with similar results. The removal rate using bursts is smaller, and the burr formation is increased compared to single pulses. These effects were confirmed by the simulations of Matsumota et al. [133]. Using a 10-pulse burst with an intra-burst delay of 16.7 ns (corresponding to an intra-burst repetition rate of 60 MHz) led to a decrease in the ablation depth of about 40% compared to single pulse operation using the same average power in the case of stainless steel [22]. The authors have attributed this decrease to plasma shielding, as the small ablation geometry (machining of deep trenches) does not allow a fast dissolution of the plasma between two pulses. In [160], a decrease in the removal rate for stainless steel (>15%) and copper (>5%) using bursts with an intra-burst delay of 1 ns (corresponding to an intra-burst repetition rate of 1 GHz) and different energy distributions was reported. Using a similar intra-burst delay of 1.13 ns (corresponding to an intra-burst repetition rate of 0.88 GHz), it was reported by Bonamis et al. that in certain conditions (high fluence and a pulse overlap higher than 70%) the grooves were refilled by molten material [135]. The same effect was also observed in line scribing using single pulse operation at a comparably high pulse repetition rate of 4.1 MHz [161]. Simulations confirm this phenomenon for GHz bursts [133]. It has also been shown that using a pulse burst consisting of 160 pulses with an intra-burst delay of 1.16 ns (corresponding to a frequency of 864 MHz) leads to a larger burr compared to single pulses [133].

Principally, no advantages in using ultra-short pulse bursts for line scribing has been reported in the literature so far. Further research is also needed to clarify the influence of pulse bursts in the field of precision cutting.

### 3.3. Surface Structures

#### 3.3.1. Polishing/Smoothing

In ultra-short pulsed laser machining of some metals like steel and titanium, it is well known that in certain conditions bumpy surfaces can appear [29,162]. In most applications, these bumps usually need to be avoided.

**Figure 13:**
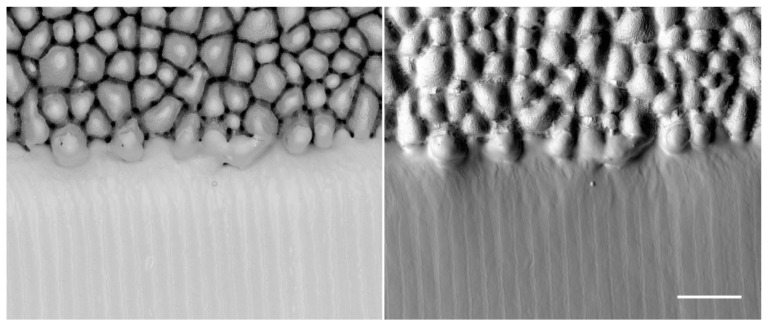
SEM images of a bumpy surface on 1.4301 (AISI 304) steel, where the lower part is polished using GHz bursts. The right-hand image shows the topological view. The length of the scale bar represents 40 µm. Reprinted from [142].

As it was found by Bauer et al., those surfaces are created if the surface temperature is higher than about 600 °C before the next laser pulse impinges on the surface in case of stainless steel 1.4301 (AISI 304) [73]. Heat accumulation is the limiting factor for the productivity of laser processes where a good surface quality is required. Alternatively, heat accumulation can also be used as an advantage. Lickschat et al. [105] and Herrmann et al. [163] have shown a smoothing of cavities using pulse bursts. Nyenhuis et al. [142] and Michalowski et al. [143] have made the same observations and have further developed a smoothing process for stainless steel 1.4301 (AISI 304). Using GHz bursts with an intra-burst delay of 625 ps (corresponding to an intra-burst repetition rate of 1.6 GHz) and a fluence for each individual pulse below the threshold fluence, the heat input into the material can be utilized to create a small melt film that smoothens the bumpy surface (cf. Figure 13).

**Figure 14:**
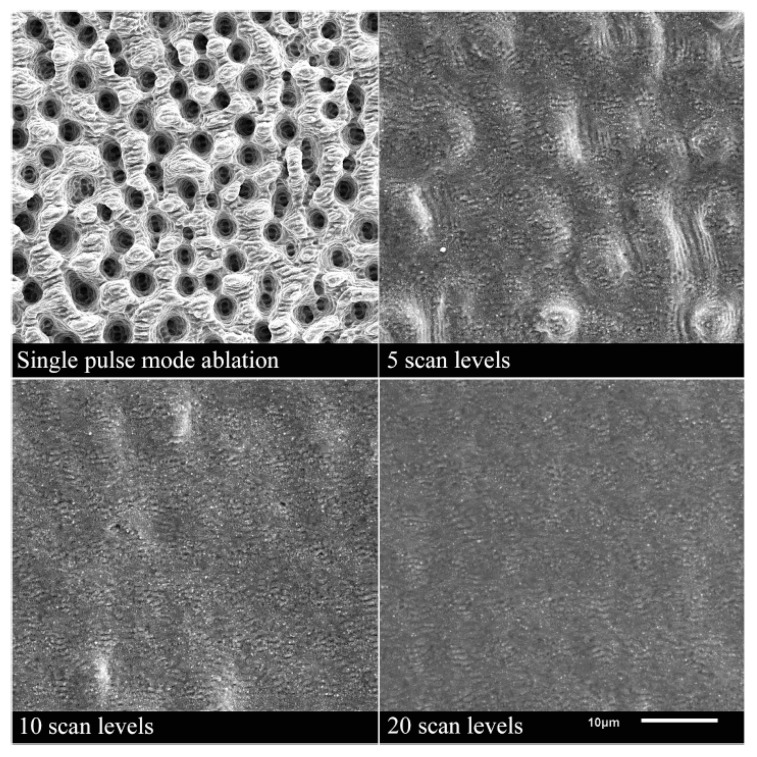
SEM images of surface structures on stainless steel. Top left: Surface structure, ablated in single pulse operation, a scan speed of 1.2 m/s, a pulse duration of 270 fs and 20 overscans (scan levels). The other SEM images visualize the structure after the surface treatment in the GHz burst mode with four pulses per burst, a pulse duration of 270 fs and a variation of the overscans (scan levels) between 5 and 20. Reprinted from [21], Copyright 2020, with permission from Elsevier.

It has been found that high fluences lead to pores in the solidified melt films. Therefore, it is favorable to use more pulses in the burst, i.e., a burst of longer time span at lower fluences of the burst pulses. In comparison to a MHz burst (intra-burst delay of 12.5 ns), the smoothing using GHz bursts is much more energy efficient as the total fluence of the burst needed is about 2/3 smaller using such GHz bursts [142]. The low fluences used in the GHz burst processing lead to a very thin melt film which can also be used for polishing of microstructures without damaging them [142,143]. Using this kind of polishing process can lead to a surface roughness of $S_{a}=$0.13 μm and $S_{z}=$ 3.4 μm [164]. Using an intra-burst delay of 200 ps (corresponding to a repetition rate of 5 GHz) Metzner et al. have shown a smoothing on stainless steel surfaces [21]. The bumpy surface created during single pulse engraving was smoothed using GHz bursts. The more layers with GHz bursts are applied to the surface, the smoother the surface gets. No bumps are present on the surface anymore after 20 overscans (scan levels), as can be seen in Figure 14. After 20 overscans, a surface roughness of $S_{a}=$ 0.1 ± 0.05 μm and $S_{z}=$ 1.3 ± 0.3 μm was measured.

To be able to use the maximum available pulse energy provided by a laser system, engraving processes can be done using MHz bursts instead of single pulse operation, followed by a GHz-polishing step. For such multi-step strategies, surface roughness values of $S_{a}=$ 0.15 ± 0.02 μm and $S_{z}=$1.1 ± 0.1 μm have been measured. Again, after 20 overscans, the surface was found to be smoothed [21]. The advantage of processes combining MHz and GHz bursts is the higher productivity, i.e., smaller cycle time during engraving processes.

In contrast, Brenner et al. used MHz bursts with an intra-burst delay $t_{B}$ of 12.5 ns (corresponding to a repetition rate of 80 MHz) at a base repetition rate $f_{rep}$ of 2 MHz (inter-burst delay of 500 ns) instead of GHz bursts to polish the surface of conducted hot-working steel (1.2738) [130]. The achieved surface roughness amounted to $S_{a}=$ 0.21 μm and $S_{z}=$ 3.65 μm. Osbild et al. analyzed the polishing process using MHz bursts in more detail [128]. It was found that using a low number of repetitions in the polishing process is favorable to achieve a melt pool depth of only 1 to 3 µm. By doing so, the roughness can be reduced also on small and fine structures.

**Figure 15:**
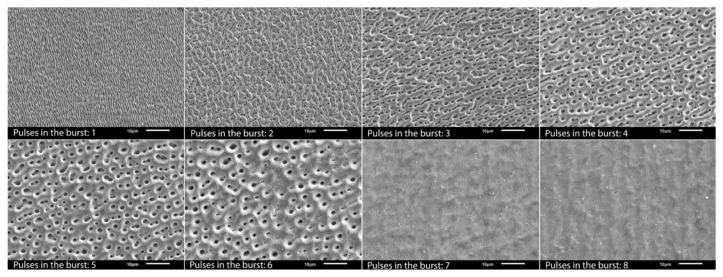
SEM images of the irradiated cobalt alloy surface as a function of the number of pulses in the burst at a fluence of 0.5 J/cm^2^ per pulse. Reproduced under the terms of a Creative Commons Attribution 4.0 International License, (https://creativecommons.org/licenses/by/4.0/ (accessed on 31 May 2021)) [132]. Copyright 2020, the authors, published by Springer Nature.

On a CoCrMo alloy, 3 to 5-pulse bursts with an intra-burst delay of 12.5 ns (corresponding to a repetition rate of 80 MHz) may lead to a much smoother surface compared to single pulse operation [78]. The surface roughness for single pulses increases with the structure depth, i.e., with increasing number of overscans, ranging from 300 nm for 10 overscans to 2.7 µm for 399 overscans. Using pulse bursts the smoothing effect can be maintained regardless of the structure depth. In another study, Metzner et al. used bursts with up to 8 pulses and an intra-burst delay of 12.5 ns (corresponding to a repetition rate of 80 MHz) and a repetition rate of 100 kHz (inter-burst delay of 10 µs) to reduce the surface roughness on cobalt and titanium alloys below $S_{a\;}=$ 100 nm [132]. Depending on the material and the used fluence of an individual pulse in the burst, different numbers of pulses in the burst (in general 6–8 pulses) need to be applied. Using a higher number of pulses in the burst also helps to avoid the formation of nanoscopic cavities on the surface as shown in Figure 15 for cobalt alloy. The same also can be observed for titanium [132].

Recently, Sassmannshausen et al. introduced the possibility of surface smoothing by continuous surface melting using a laser emitting ultra-short pulses at a pulse repetition rate of 49 MHz (corresponding to an inter-pulse delay of 20.4 ns). This extreme case on one hand represents classical repetitive processing at a constant repetition rate, while on the other hand it can be interpreted as a very long burst pulse in the MHz regime. This continuous processing shows the capability of long, finely tuned burst pulses. It was shown that the surface roughness could be lowered by this polishing process to around 80 nm. While also an oxidation of the surface was introduced, this value belongs to the smallest ever achieved by laser surface processing [165].

In a comprehensive study, Metzner et al. compared the smoothing effects using a MHz burst, a GHz burst, and a biburst (a combination of MHz and GHz bursts) on stainless steel [131]. It was found that the smoothing process shows a higher stability and reproducibility when using GHz bursts compared to MHz bursts. A surface roughness of $S_{a}<$ 200 nm was measured for processing with the GHz burst in optimal conditions. It should be mentioned that not only metals but also polymers can be polished applying pulse bursts with an intra-burst delay of 400 ps (corresponding to an intra-burst repetition rate of 2.5 GHz) [166].

#### 3.3.2. Coloring

**Figure 16:**
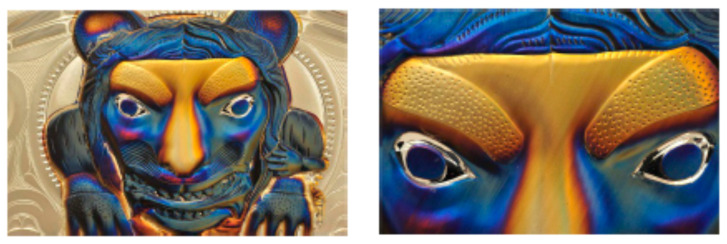
Photograph of a colored silver coin (height approx. 1 cm); detailed view on the right-hand side. Reproduced under the terms of a Creative Commons Attribution 4.0 International License (https://creativecommons.org/licenses/by/4.0/ (accessed on 31 May 2021)) [175]. Copyright 2017, the authors, published by Springer Nature.

Laser coloring was intensively investigated for ns pulse processing where oxide layers are formed [167,168,169,170]. Alternatively, ultra-short pulsed lasers can be used. This is mainly realized by the following approaches: utilizing laser-induced periodic surface structures (LIPSS) as diffraction gratings to produce angle-sensitive colors [171,172] and creating tempering colors by heating up the surface [173], which results in angle-independent colors. Another way is to create micro- and nanostructures on the surface to obtain a certain color on the surface [171,174]. The coloring strategy using nanostructures was further developed by Guay et al. [175]. Plasmonic colors are excited from metallic nanoparticles and nanostructures, which are re-deposited after the laser irradiation leads to angle-independent colors on silver surfaces (cf. Figure 16).

**Figure 17:**
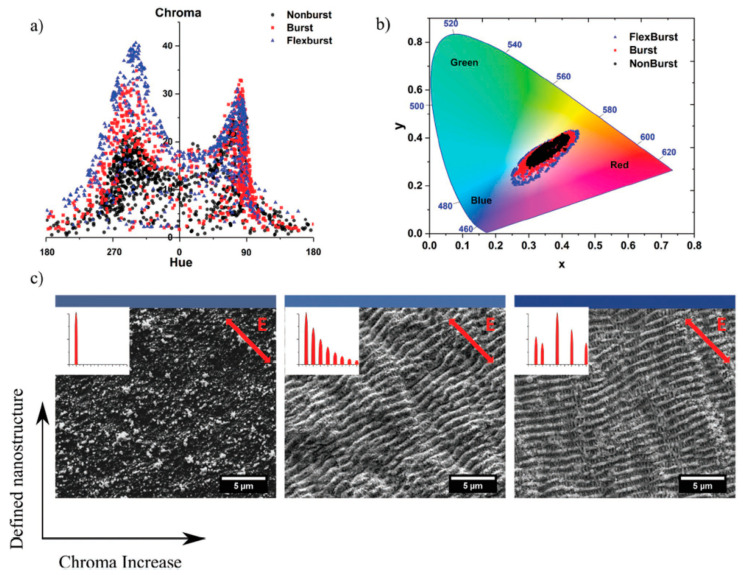
Colors and corresponding topographies on a silver surface. (**a**) Graph of Chroma versus hue comparing colors obtained after single pulse operation (●), burst (■), and flexburst (▴) coloring. (**b**) CIE *xy* chromaticity diagram comparing single pulse operation (●), burst (■), and flexburst (▴) coloring. (**c**) SEM images of blue surfaces produced using single pulse operation (**left**), burst (**middle**), and flexburst (**right**) coloring. The hue was more or less the same for all squares (H ≈ 295°), whereas the Chroma values equalled 22.3, 31.2, and 39.44. Nanostructures can be observed on the surfaces for burst and flexburst processing. The relative energy distribution of the burst pulses and the orientation of the electric field are given as insets. Reprinted from [178], Copyright Wiley-VCH GmbH. Reproduced with permission.

It was shown that by using pulse bursts with an intra-burst delay of 12.8 ns (corresponding to an intra-burst repetition rate of 82 MHz), higher quality colors can be generated on silver compared to a single pulse operation. The authors quantified the colors using a Chroma meter (CR-241, Konica Minolta) in the CIELCH color space. The Chroma is a parameter used to measure the colorfulness of an object. The Chroma is increased by about 50% over the full color range when processing with burst pulses compared to colors produced with single pulses [176]. The increase in Chroma using bursts can be explained by the simultaneous creation of LIPSS onto which the nanoparticles are re-deposited, compared to single pulses where no LIPSS are formed (cf. Figure 17c). The increase in the surface area created by the LIPSS and the field enhancement in these crevices would cause higher absorption, explaining the more vibrant colors unique to the burst process. In addition, a larger color palette can be created using pulse bursts. By using the FlexBurst^TM^ technology of the laser manufacturer Lumentum (formerly Time-Bandwidth Products, Zurich, Switzerland) [177], it is possible to fade out some pulses in the burst, i.e., adapting the intra-burst delay in steps of 12.8 ns and also adapting the pulse energy of every individual pulse within the burst. This modification of the bursts leads to a further increase in Chroma (Figure 17a) and a larger color palette (Figure 17b) [178]. The developed strategy was applied on the metals: gold, silver, copper, and aluminum.

#### 3.3.3. Laser-Induced Periodic Surface Structures (LIPSS)

Laser-induced periodic surface structures, often also called ripples, can be applied on almost every material surface using polarized light. Usually, linearly polarized light is used to create such structures. Applications for LIPSS can be found in various fields. The surfaces appearing in nature (colored surfaces, anti-reflection surfaces, hydrophobic surfaces, wet and dry adhesion, and friction reduction) can be imitated [179] or the melt flow resistance in injection molding of polypropylene can be reduced [180]. LIPSS can be replicated as well on plastic parts [181,182]. The phenomena behind the creation of ripples is discussed in several publications [183,184,185,186]. In the last years, multiple investigations using pulse bursts have been performed with different polarizations of the pulses in double pulses [187], different wavelengths in double pulses [188], and with variations in the energy distribution [189,190]. In addition, the number of pulses in the burst has been varied by up to 32 pulses [191].

**Figure 18:**
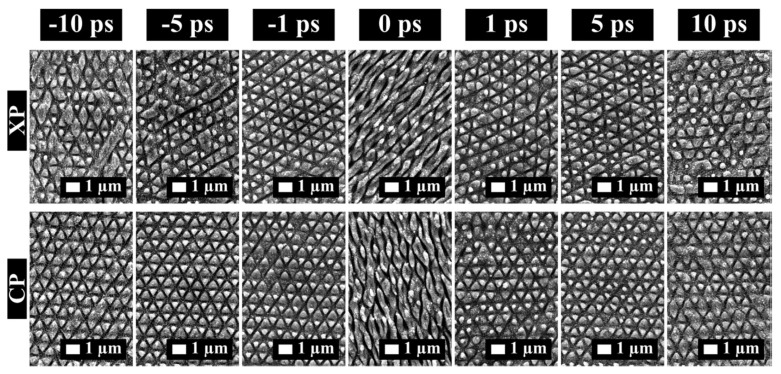
SEM images of the surface structures on stainless steel irradiated with two different polarization configurations: cross-polarized double pulses (XP) and counter-rotating circular-polarized pulses (CP). The rest of the process parameters were fixed: 10 pulses per spot, hatch distance of 1 µm, and a fluence of 0.1 J/cm^2^. The delay of the two pulses was varied as indicated on top of the images. Reprinted from [187], copyright 2019, with permission from Elsevier.

Combining two time-delayed ultra-short pulses with different polarization states can generate different structure types than conventional LIPSS, which are formed parallel to the polarization direction for linear polarization [187]. Figure 18 shows the variations of the surface structures produced by double pulses with delays of up to 10 ps (corresponding to an intra-burst repetition rate of 100 GHz) and either cross-polarized (XP) or counter-rotating circular-polarized (CP) pulses.

Increasing the number of pulses in the burst from 2 to 32 pulses while maintaining the intra-burst delay of 1.5 ps (corresponding to a repetition rate of 666 GHz) and linear polarization leads to an increase in the spatial period of the low spatial frequency LIPSS on stainless steel, while the depth of the LIPSS remains almost constant [191]. It should be mentioned that the spatial period of multiple pulses is always larger than for single pulse operation. Increasing the intra-burst delay from 1.5 ps to 24 ps using double pulses also leads to an increase in the spatial frequency, while the depth of the structures decreases drastically. This decrease is attributed to shielding effects taking place for delays larger than about 6 ps [191].

Using cross-polarized light instead of the parallel polarization for all pulses in the burst will lead to similar structures, as shown in Figure 18 [192]. Comparing the wettability of triangular structures produced with a four-pulse burst at an intra-burst delay of 1.5 ps (corresponding to an intra-burst repetition rate of 666 GHz) with normal LIPSS created with a 16-pulse burst and an identical intra-burst delay shows only a difference in the evolution of the water contact angle over time, known as the aging effect, for the two different surface structures. The final water contact angle, which is reached after about eight weeks, was principally the same. It can be concluded that not only does the surface structures have an influence on the wettability but also the surface chemistry [192].

Another approach is to combine two cross-polarized pulses with different fluences. The LIPSS’ orientation on a titanium substrate can be influenced by the fluence of the delayed pulse and is in good agreement with the direction obtained by the vector sum of the laser fields. For example, using two pulses with polarizations perpendicular to each other with the same pulse energy, the LIPSS are formed at an angle of 45 degrees, while when one pulse has half the energy of the other pulse, LIPSS are formed at an angle of 24.5 degrees [190]. A similar setup but with equal polarization states for both pulses and a fixed intra-burst delay of 160 fs (corresponding to a repetition rate of 6.25 THz) was used by Hashida et al. [189]. The fluence of the first pulse was always below the threshold fluence, whereas the fluence of the delayed pulse was above the threshold fluence. It was shown that the laser fluence of the first pulse plays an important role for the spacing of the LIPSS. The higher the fluence of the first pulse, the larger the spacing becomes. Comparing the grating spacing produced with double pulses with the one produced with single pulses with the same total energy shows no difference [189]. Larger intra-burst delays of 20 ns (corresponding to a repetition rate of 50 MHz) have been used by Wang et al. [193] together with a higher number of pulses. Depending on the used fluence of an individual pulse in the burst, either low spatial frequency LIPSS (LSFL) or high spatial frequency LIPSS (HSFL) are formed on the surface. For a fixed total fluence of the burst of 0.25 J/cm^2^, the formation of HSFL is enhanced by using three or four pulses in the burst, as the fluence of each pulse in the burst becomes smaller. Increasing the total fluence will lead to microgrooves, nanoholes, and melt formations in the central region of the machined grooves, sometimes referred to as “micro-hills” [194].

The use of double pulses with different wavelength (800 nm and 400 nm), fluences, and intra-burst delays and the use of cross-polarization states was investigated by Hashida et al. [188]. It was found that the fundamental pulse (800 nm) is responsible for the LIPSS creation and its orientation, while the second harmonic pulse will optimize the uniformity of the LIPSS if intra-burst delays of 0 to 2 ps are used. For delayed pulses (first pulse with 400 nm and the second pulse with 800 nm), the period and the direction of the ripples can be changed [188].

Experiments investigating the influence of double pulses onto the LIPSS formation have also been performed on semiconductors and glasses but are not further discussed in this article [195,196].

### 3.4. Milling

**Figure 19:**
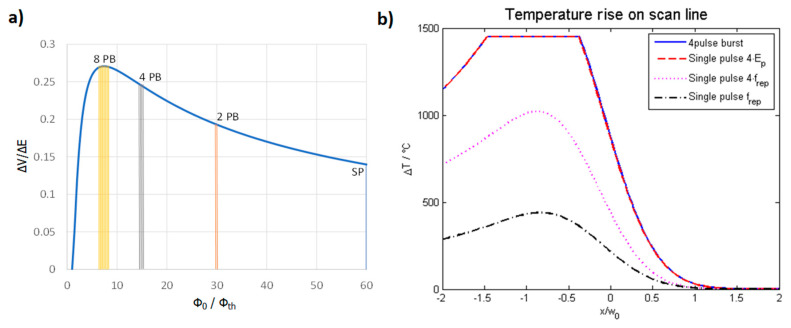
(**a**) Normalized energy specific volume as a function of the normalized peak fluence and the corresponding energy specific volumes for a total fluence of 60 times the threshold fluence for single pulses, double pulses, and bursts consisting of 4 and 8 pulses. (**b**) Temperature rise on the scan line just before the next burst sequence impinges on the surface for a 4-pulse burst on steel 1.4301 (AISI 304) with 12 W average power, 500 kHz repetition rate, and a pitch of 8 µm. The temperature rise is plotted for a burst with 4 pulses (blue line), single pulses with 4 times higher pulse energy (red dashed line), single pulses with 4 times higher repetition rate of 2 MHz (magenta dotted line), and single pulses having the same energy at a repetition rate of 500 kHz (black dash-dotted line). The plateau denotes the region where melting happens. The distance x is given in units of the spot radius w, and, as the previous pulse has struck the surface at x=−0.5·w, the maximum temperatures appears around this region. (**b**) was reprinted from [14].

The basic benefit of the burst mode for milling applications is illustrated by the following consideration: milling applications are generally realized with galvanometric scanners offering maximum marking speeds of a few tens of m/s [197]. The pulse to pulse distance, i.e., the pitch, is defined by the repetition rate of the laser and the marking speed of the scanner. Thus, the maximum scanner speed limits the applicable laser repetition rate if a certain minimum value of the pitch has to be kept. Therefore, often pulse energies and peak fluences far above the optimum value are used to take advantage of the full average power of the used laser system. At this high fluence, the energy specific volume is significantly lower than its maximum value, as illustrated in Figure 19a, for single pulses having a peak fluence of 8 times the optimum value. Dividing this energy into n sub-pulses, i.e., into an n-pulse burst, reduces the fluence of these sub-pulses and shifts the energy specific volume for each pulse within the burst nearer to its optimum value (cf. Figure 19a). Additionally, following Figure 5, the ablation process is moved from the phase explosion into the spallation regime and an improved surface quality can be expected. It has to be noted that the heat accumulation is only slightly affected. Calculations for steel 1.4301 (AISI 304) [14] following the analytical model presented in [73] reveal that the temperatures just before the next burst sequence impinges on the surface do not differ between a four-pulse burst and single pulses with four times higher pulse energy (cf. Figure 19b).

**Figure 20:**
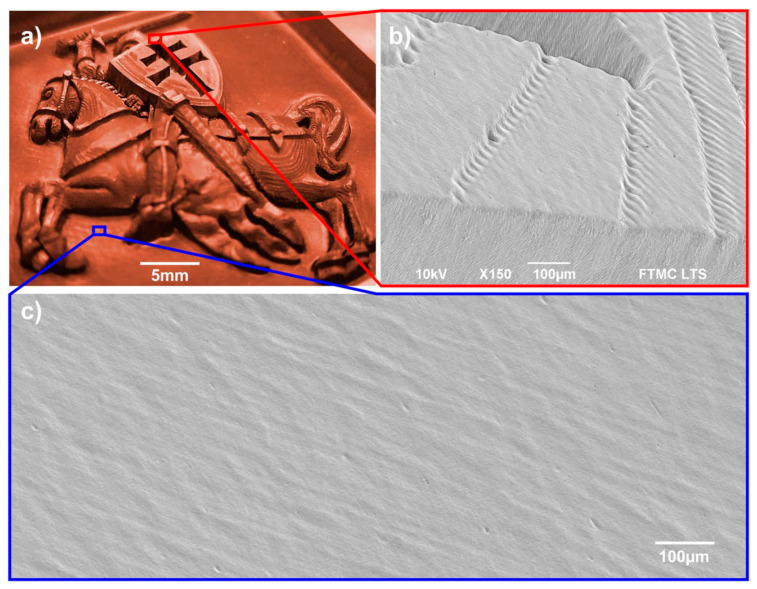
Example of efficient laser milling. (**a**) Optical image of the coat of arms of Lithuania milled in a copper plate. (**b**) SEM image of laser-milled surface illustrating layer-by-layer removal. (**c**) SEM image of the bottom surface of the laser-milled cavity. Laser parameters—triple pulse, laser wavelength of 1030 nm, burst repetition rate 300 kHz, intra-burst repetition rate of 64.5 MHz, and beam scanning speed of 1 m/s. Reproduced under the terms of a Creative Commons Attribution 4.0 International License, (https://creativecommons.org/licenses/by/4.0/ (accessed on 31 May 2021)) [18]. Copyright 2019, the authors, published by Springer Nature.

The machining of stainless steel in single pulse operation is only possible in the low fluence regime, as the hole and spike formation limit the surface quality and therefore the productivity at higher fluences [14,198]. Using MHz bursts, the fluence of an individual pulse in the burst can be above the optimal fluence, as the bursts help to avoid this hole formation and therefore are more beneficial [60]. As shown in Section 2.3., machining using a triple pulse on copper can be more efficient than using single pulse operation. A milling result using this strategy is shown in Figure 20.

It should be mentioned here that, to optimize the productivity in ultra-short pulsed laser milling, not only the ablation process itself must be optimized but also the scan strategy. It has been shown that by using optimized scan speeds, the time for machining a certain geometry, e.g., a line, can be optimized in the case of galvanometer scanners [197]. The given values of the energy specific volume are for 100% laser-on time, which is not true for galvanometer-based scanning using the sky-writing mode, which is used to obtain the best machining quality, i.e., no over-engraving near the edges [199]. The optimization of the energy specific volume alone makes no sense if the duty cycle of the scanner is near zero in the final application.

**Figure 21:**
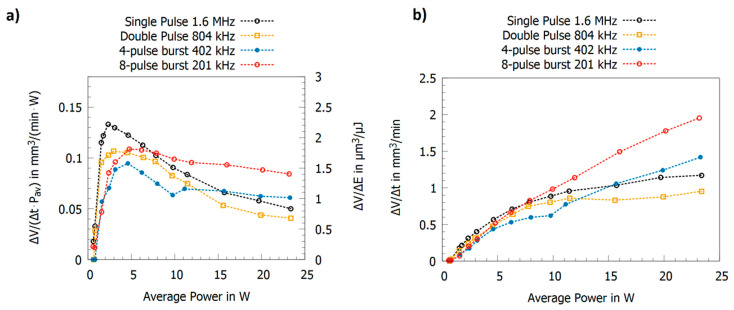
(**a**) Energy specific volumes and (**b**) removal rates for steel 1.4301 (AISI 304) machined with pulses of 10 ps pulse duration with a wavelength of 1064 nm and a spot radius of 15.5 µm. To guarantee identical peak fluence of the individual pulses, the laser repetition rate was 1610 kHz for single pulses, 804 kHz for the double pulse burst, 402 kHz for the burst with four pulses, and 200 kHz for the burst with eight pulses. Data extracted from [14].

The former considerations are based on the assumption that only the peak fluence of the single pulses in a burst defines the energy specific volume and that the latter does not depend on the number of pulses per burst. This is definitively not the case, as, e.g., shown in [14,130] for stainless steel and as discussed in Section 2.3 and Section 2.4, as the number of pulses as well as the intra-burst delay affect the energy specific volume. Further, to compare situations with equal average power single pulse operation, a repetition rate $f_{rep}$ should be compared with a n-pulse burst with a repetition rate of $f_{rep}/n$. In Figure 21, based on the results for stainless steel 1.4301 (AISI 304) machined with 10 ps pulse duration at a wavelength of 1064 nm and a spot radius of 15.5 µm [14], the energy specific volumes as well as the removal rates are shown for bursts consisting of eight, four, two, and one pulses at corresponding repetition rates of 200 kHz, 402 kHz, 804 kHz, and 1610 kHz, respectively. Up to an average power of about 7.5 W, the energy specific volume for single pulses at a repetition rate of 1610 kHz exceeds that of the burst with eight pulses, and the corresponding removal rates are higher for single pulses. For higher average powers, the situation changes, and bursts with eight pulses become more efficient, while the removal rate exceeds that of all other presented situations. These effects could eventually be caused by the smoothing effect and the reduction in the surface structures as shown in Figure 13, Figure 14 and Figure 15.

The situation may change when higher repetition rates are used for single pulses, e.g., by using polygon line scanners, which can now achieve marking speeds of several 100 m/s [200,201]. However, the scale-up process is limited either by heat accumulation, especially for steel, and/or shielding effects discussed in the double pulse section, as shown in [202].

**Figure 22:**
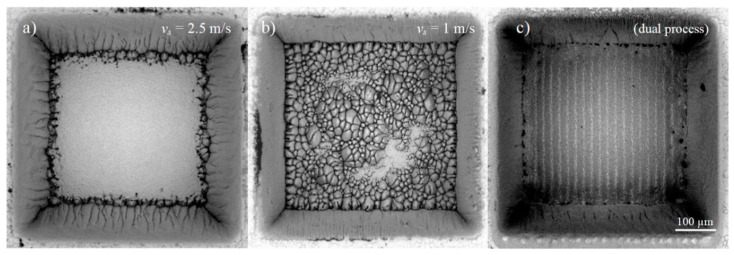
SEM images of milled squares with a scan speed of (**a**) 2.5 m/s and (**b**) 1 m/s at a conventional process and 1 m/s for (**c**) using the dual process strategy. The surface roughness of the inner ground was $S_{a}=$ 0.23 μm and $S_{z}=$ 4.1 μm for (**a**), but was not measurable because of high occurrence of bumps for (**b**); and $S_{a}=$ 0.13 μm and $S_{z}=$ 3.4 μm for (**c**). Although the scanning speed equaled that of (**b**), the edge and surface quality was the best and just the line spacing during polishing limited the relatively high $S_{z}$ value. Reproduced from ref. [164] with permission from Japan Laser Processing Society.

Therefore, for concrete applications, an optimization of processing strategies with respect to the number of pulses per burst, the laser repetition rate, the spot size, and the machining quality is demanded to find the best-suited parameters. Dual process strategies using different parameter sets, e.g., one for roughing with high energies and high removal rates followed by one with pulse bursts, either in the MHz or even the GHz regime used for finishing/polishing are very promising. For example, a dual process strategy of alternating GHz bursts and conventional processing was presented to achieve a good surface quality of machined pockets while decreasing the processing time by a factor of two, as illustrated in Figure 22.

Brenner et al. [203] demonstrated another interesting application by combining ablation processing in burst mode followed by a cleaning and an additional polishing process for large-format 3D mold tools. Another new approach of combining MHz and GHz bursts, the bi-burst mode, was recently presented in [24,131], but, in milling applications for copper and steel, this mode did not reveal higher removal rates compared to single pulses. In the field of laser burst processing, a comparison of literature data regarding roughness and energy specific volumes for different metals is a challenging task due to different study designs. For a comprehensive comparison of data for at least the metals of copper and stainless steel, the reader may refer to [24].

To conclude, the combination of different processes taking benefit of the advantages of each milling process to obtain optimum quality and machining time when using MHz and GHz bursts is a widely open field and still demands further research.

### 3.5. Further Applications

Besides the micromachining of metals in the industrial environment, pulse bursts are also used in several other fields. As a completion, these topics are briefly introduced in the following subsections.

#### 3.5.1. Laser-Ablative Space Propulsion

Another possible application of ultra-short laser pulses is the propulsion of either space vessels or space debris. When irradiating material and inducing ablation, the sample experiences a small force in the range of nN or even µN. The ablated material serves as propellant material. This force can be used as thrust to either stabilize and correct the orbits of space vessels, such as satellites, or to decelerate space debris in order to clean the earth’s orbit [204,205,206,207,208,209,210]. Recently, it was shown that using ultra-short double pulses can give added value to this specific application. When using double pulses, material is pushed back to the initial surface. It was shown that, on the one hand, the measured thrust increases by a factor of two when using a double pulse instead of a single pulse of the same total energy (or using the same average power for processing) [114]. On the other hand, since the material is pushed back and can be used as propellant again, the thrust per ablated mass increases by a factor of three, leading to a more efficient propulsion and propellant conservation at the same time [114].

#### 3.5.2. Laser-Induced Breakdown Spectroscopy

Since ablated material is able to shield subsequent laser pulses, a specific technique allowing the characterization of matter, laser-induced breakdown spectroscopy, can profit from the atomization of the shielding matter due to the emittance of characteristic spectra. LIBS relies on spectra emitted from the vapour and plasma created after a laser pulse hits a material surface. Either the occurrence or the intensity of specific characteristic emission lines as well as the ratios of such lines is investigated to gain information about the material. This characterization technique principally goes back to the early years of the laser in the 1960s after the first demonstration of Q-Switch lasers, and, until the end of the 20th century, it was basically performed using lasers with nanosecond pulse durations [211,212,213]. In 1998, the first LIBS measurements were reported using ultra-short pulsed lasers [214], and it was found that the ablation process itself and the atom excitation is more reproducible for fs than for ns pulses [215]. Hence, less energy is needed for excitation, which is crucial for the characterization of specific samples in order to keep the destruction of those limited, e.g., when examining paintings [216,217,218] or explosives [219,220,221]. Additionally, the sensitivity is higher when using fs lasers [215]. Several summarizing papers can be found in the literature regarding the single pulse fs-LIBS technique [222,223,224]. Signal enhancement of the observed spectra can be obtained when using double pulses or multiple burst pulses [7,91,223,225,226], since the atomistic and plasma excitation and resulting temperatures are much higher compared to single pulses, as has been discussed before. While material processing scientists may suffer from ablation suppression when using double pulses, spectroscopists look forward to the enhanced excitation of matter due to the atomization of the shielding material.

#### 3.5.3. Generation of Particle Beams and X-rays

Ultra-short laser pulses can also be used for the generation of beams consisting of particles such as protons, electrons, and heavy ions as well as X-rays [227,228,229,230,231,232,233,234,235,236,237] due to the excitation of matter and the generation of high-energy plasmas. The range of radiation that can be generated is already extensive, but the range of applications is even wider and includes medical applications in radiology and radiotherapy [227,238,239,240]. Moreover, characterization of matter can be performed using a variety of investigation methods based on particle beams [233,235,236]. Several aspects, such as generated intensity, quality and purity of the spectra, as well as well-defined energies of the created radiation, are important for such applications and rely highly on the correct excitation of matter.

Double pulses (sometimes referred to as pre-pulses in this specific field of particle science) allow a specific manipulation of the excitation states of warm matter. The pulse delays lie in the range of the pulse duration (several 10 to 100 fs) and up to several ps and allow the energy and spectrum of highly charged silver ions [241], the energy and spectrum of created protons [231,242], and the spectrum of created X-rays to be tuned [243].

## 4. Conclusions

Metal processing using ultra-short burst pulses has been an emerging field in laser processing in the past decade and is still a subject for further investigation. In this review article, the physical mechanisms involved during laser burst processing were summarized. Several aspects such as the shielding of radiation, the re-deposition of material, enhanced absorptance, and heat accumulation have to be considered when designing ablation processes as the intra-burst delay is shortened and the number of pulses within bursts is increased. Although the occurring phenomena are widely understood for double pulses, the involved phenomena being able to explain the measured removal rates and energy specific volumes for triple and multi-pulse burst ablation for different metals are not understood to a large extent. Therefore, research in this field is still needed.

Re-deposition of material can be utilized for surface polishing when using burst pulses in the MHz regime for processing. It allows a smoothing of material surfaces with a low number of burst pulses due to re-deposition of material in the liquid state. For higher pulse numbers within a burst, heat accumulation enhances this effect and can lead to stable melt layers on the surface during processing. If the intra-burst repetition rate is increased to several GHz or more, heat accumulation effects can be utilized to smooth the surface (for lower pulse energies) and to enhance ablation (for higher pulse energies) in the sense that it becomes as effective as ns laser processing. The increase in ablated volume per energy and per time is accompanied by a higher surface roughness, which fits the effects known from ns laser ablation.

The occurring physical mechanisms allow a variety of innovative applications, including surface smoothing, plasmonic coloring of surfaces, and, in general, an optimum distribution of energy in order to completely utilize the average power of ultra-short pulsed lasers. The future belongs to laser systems that allow a variety of burst pulse combinations with intra-burst repetition rates in the MHz and the GHz regimes, allowing a realization of combined processes on the machined workpiece.

## Figures and Tables

**Table 1:** Electron–phonon coupling times for various metals at room temperature.

Material	Ag	Al	Au	Cu	Fe	Mb	Ni	Pt	Ti	W
*τ_el-phon_* in ps	84.3 ^1^	4.5 ^1^	115.5 ^1^	57.5 ^2^	1.3 ^3^	2.2 ^4^	1.1 ^4^	2.2 ^5^	1.9 ^5^	12.1 ^5^

^1^ calc. from *C_i_* [48], *g* [49]; ^2^ [50]; ^3^ calc. from *C_l_* [48], *γ* [51]; ^4^ calc. from *C_l_* [48], *γ* [52]; ^5^ calc. from *C_l_* [48], *γ* [53].

**Table 2:** Material parameters corresponding to the plots in Figure 5. Some of the fit parameters were calculated from original data.

Quantity		Brass ^1^	Copper ^1^	Copper ^2^	Gold ^2^	Silver ^2^	Steel ^1^	Steel ^2^
λ	nm	1030	1030	1064	1064	1064	1030	1064
τ	ps	0.4	0.4	10	10	10	0.4	10
δ (fit)	nm	62.3	55.7	29.1	64.8	67.2	13.9	4.5
$\phi_{th}$(fit)	J/cm^2^	0.39	0.49	0.35	0.35	0.36	0.09	0.06
$\Delta V/\Delta E_{max}$	mm^3^/W/min	0.26	0.19	0.14	0.30	0.30	0.25	0.13
$\Delta V/\Delta E_{max}$	µm^3^/µJ	4.3	3.1	2.3	5.0	5.0	4.2	2.2
$\phi_{0,opt}$	J/cm^2^	2.7	4.5	2.2	2.4	2.7	0.5	0.5

^1^ [60], ^2^ [59].

## Data Availability

No new data were created or analyzed in this study. Data sharing is not applicable to this article.

## References

[1] Negel J.-P., Voss A., Ahmed M.A., Bauer D., Sutter D., Killi A., Graf T. (2013). 11 kW average output power from a thin-disk multipass amplifier for ultrashort laser pulses. Opt. Lett..

[2] Negel J.-P., Loescher A., Bauer D., Sutter D., Killi A., Ahmed M.A., Graf T. (2016). Second Generation Thin-Disk Multipass Amplifier Delivering Picosecond Pulses with 2 kW of Average Output Power. Advanced Solid State Lasers, Proceedings of the Lasers Congress 2016 (ASSL, LSC, LAC), Boston, MA, USA, 30 October–3 November 2016.

[3] Nubbemeyer T., Kaumanns M., Ueffing M., Gorjan M., Alismail A., Fattahi H., Brons J., Pronin O., Barros H.G., Major Z. (2017). 1 kW, 200 mJ picosecond thin-disk laser system. Opt. Lett..

[4] Gaida C., Gebhardt M., Heuermann T., Stutzki F., Jauregui C., Limpert J. (2018). Ultrafast thulium fiber laser system emitting more than 1 kW of average power. Opt. Lett..

[5] Schnitzler C., Mans T.G., Dolkemeyer J., Dittmann P. (2019). High Power, High Energy, and High Flexibility: Powerful Ultrafast Lasers Based on InnoSlab Technology. High-Power Laser Materials Processing: Applications, Diagnostics, and Systems VIII, Proceedings of the SPIE LASE, 2019, San Francisco, CA, USA, 2–7 February 2019.

[6] Müller M., Aleshire C., Klenke A., Haddad E., Légaré F., Tünnermann A., Limpert J. (2020). 104 kW coherently combined ultrafast fiber laser. Opt. Lett..

[7] Semerok A., Dutouquet C. (2004). Ultrashort double pulse laser ablation of metals. Thin Solid Films.

[8] Donnelly T., Lunney J.G., Amoruso S., Bruzzese R., Wang X., Ni X. (2009). Double pulse ultrafast laser ablation of nickel in vacuum. J. Appl. Phys..

[9] Povarnitsyn M.E., Itina T.E., Khishchenko K.V., Levashov P.R. (2009). Suppression of Ablation in Femtosecond Double-Pulse Experiments. Phys. Rev. Lett..

[10] Povarnitsyn M.E., Fokin V.B., Levashov P.R., Itina T.E. (2015). Molecular dynamics simulation of subpicosecond double-pulse laser ablation of metals. Phys. Rev. B.

[11] Hartmann C., Gillner A., Aydin U., Noll R., Fehr T., Gehlen C., Poprawe R. (2007). Investigation on laser micro ablation of metals using ns-multi-pulses. J. Physics Conf. Ser..

[12] Hu W., Shin Y.C., King G. (2009). Modeling of multi-burst mode pico-second laser ablation for improved material removal rate. Appl. Phys. A.

[13] Hernandez-Rueda J., Siegel J., Galvan-Sosa M., De La Cruz A.R., Solis J. (2013). Surface structuring of fused silica with asymmetric femtosecond laser pulse bursts. J. Opt. Soc. Am. B.

[14] Neuenschwander B., Kramer T., Lauer B., Jaeggi B. (2015). Burst Mode with Ps- and Fs-Pulses: Influence on the Removal Rate, Surface Quality, and Heat Accumulation. Laser Applications in Microelectronic and Optoelectronic Manufacturing (LAMOM) XX, Proceedings of the SPIE LASE, San Francisco, CA, USA, 9–12 February 2015.

[15] Kerse C., Kalaycıoglu H., Elahi P., Cetin B., Kesim D.K., Akcaalan O., Yavas S., Asik M.D., Oktem B., Hoogland H. (2016). Ablation-cooled material removal with ultrafast bursts of pulses. Nature.

[16] Jäggi B., Förster D.J., Weber R., Neuenschwander B. (2018). Residual heat during laser ablation of metals with bursts of ultra-short pulses. Adv. Opt. Technol..

[17] Neuenschwander B., Jaeggi B., Foerster D.J., Kramer T., Remund S. (2019). Influence of the burst mode onto the specific removal rate for metals and semiconductors. J. Laser Appl..

[18] Zemaitis A., Gecys P., Barkauskas M., Raciukaitis G., Gedvilas M. (2019). Highly-efficient laser ablation of copper by bursts of ultrashort tuneable (fs–ps) pulses. Sci. Rep..

[19] Bornschlegel B., Finger J. (2019). In-Situ Analysis of Ultrashort Pulsed Laser Ablation with Pulse Bursts. JLMN.

[20] Gaudiuso C., Kämmer H., Dreisow F., Ancona A., Tünnermann A., Nolte S., Heisterkamp A., Herman P.R., Meunier M., Nolte S. (2016). Ablation of Silicon with Bursts of Femtosecond Laser Pulses. Frontiers in Ultrafast Optics: Biomedical, Scientific, and Industrial Applications XVI, Proceedings of the SPIE LASE, San Francisco, CA, USA, 13–18 February 2016.

[21] Metzner D., Lickschat P., Weißmantel S. (2020). High-quality surface treatment using GHz burst mode with tunable ultrashort pulses. Appl. Surf. Sci..

[22] Hodgson N., Allegre H., Starodoumov A., Bettencourt S. (2020). Femtosecond Laser Ablation in Burst Mode as a Function of Pulse Fluence and Intra-Burst Repetition Rate. JLMN.

[23] Hodgson N., Steinkopff A., Heming S., Allegre H., Haloui H., Lee T.S., Laha M., van Nunen J. (2021). Ultrafast Laser Machining: Process Optimization and Applications. Laser Applications in Microelectronic and Optoelectronic Manufacturing (LAMOM) XXVI, Proceedings of the SPIE LASE, San Francisco, CA, USA, 6–12 March 2021.

[24] Žemaitis A., Gaidys M., Gečys P., Barkauskas M., Gedvilas M. (2021). Femtosecond laser ablation by bibursts in the MHz and GHz pulse repetition rates. Opt. Express.

[25] Führa B., Russ S., Hammers-Weber P., Diego-Vallejo D., Kahmann M., Andreev A., Hesse T. High precision drilling with ultra-short laser pulses. Proceedings of the Lasers in Manufacturing Conference 2017.

[26] Žemaitis A., Gaidys M., Brikas M., Gečys P., Raciukaitis G., Gedvilas M. (2018). Advanced laser scanning for highly-efficient ablation and ultrafast surface structuring: Experiment and model. Sci. Rep..

[27] Jaeggi B., Neuenschwander B., Meier T., Zimmermann M., Hennig G. (2013). High Precision Surface Structuring with Ultra-Short Laser Pulses and Synchronized Mechanical Axes. Phys. Procedia.

[28] Kramer T., Remund S., Gafner M., Zwygart D., Neuenschwander B., Holtz R., Witte R., Dury N. (2018). Novel strategy for ultrafast pulsed laser micromachining of rotational symmetric metallic parts. Procedia CIRP.

[29] Bruening S., Hennig G., Eifel S., Gillner A. (2011). Ultrafast Scan Techniques for 3D-μm Structuring of Metal Surfaces with high repetitive ps-laser pulses. Phys. Procedia.

[30] Liu X., Du D., Mourou G. (1997). Laser ablation and micromachining with ultrashort laser pulses. IEEE J. Quantum Electron..

[31] Shirk M.D., Molian P.A. (1998). A review of ultrashort pulsed laser ablation of materials. J. Laser Appl..

[32] Sundaram S.K., Mazur E. (2002). Inducing and probing non-thermal transitions in semiconductors using femtosecond laser pulses. Nat. Mater..

[33] Perry M.D., Stuart B.C., Banks P.S., Feit M.D., Yanovsky V., Rubenchik A.M. (1999). Ultrashort-pulse laser machining of electric materials. J. Appl. Phys..

[34] Krüger J., Kautek W. (2012). Ultrashort Pulse Laser Interaction with Dielectrics and Polymers. Advances in Polymer Science.

[35] Bulgakova N.M., Zhukov V.P., Collins A.R., Rostohar D., Derrien T.J.-Y., Mocek T. (2015). How to optimize ultrashort pulse laser interaction with glass surfaces in cutting regimes?. Appl. Surf. Sci..

[36] Samant A.N., Dahotre N.B. (2009). Laser machining of structural ceramics—A review. J. Eur. Ceram. Soc..

[37] Chichkov B.N., Momma C., Nolte S., von Alvensleben F., Tünnermann A. (1996). Femtosecond, picosecond and nanosecond laser ablation of solids. Appl. Phys. A.

[38] Cheng J., Liu C., Shang S., Liu D., Perrie W., Dearden G., Watkins K. (2013). A review of ultrafast laser materials micromachining. Opt. Laser Technol..

[39] Mishra S., Yadava V. (2015). Laser Beam MicroMachining (LBMM)—A review. Opt. Lasers Eng..

[40] Lei S., Zhao X., Yu X., Hu A., Vukelic S., Jun M.B.G., Joe H.-E., Yao Y.L., Shin Y.C. (2020). Ultrafast Laser Applications in Man-ufacturing Processes: A State-of-the-Art Review. J. Manuf. Sci. Eng..

[41] Momma C., Nolte S., Chichkov B., Tunnermann A., Von Alvensleben F. Precise Laser Ablation with Ultra-Short Pulses. Proceedings of the European Meeting on Lasers and Electro-Optics CLEOE-96.

[42] Zhigilei L. (2003). Dynamics of the plume formation and parameters of the ejected clusters in short-pulse laser ablation. Appl. Phys. A.

[43] Ivanov D.S., Zhigilei L. (2003). Combined atomistic-continuum modeling of short-pulse laser melting and disintegration of metal films. Phys. Rev. B.

[44] Povarnitsyn M.E., Andreev N.E., Apfelbaum E.M., Itina T.E., Khishchenko K., Kostenko O.F., Levashov P.R., Veysman M.E. (2012). A wide-range model for simulation of pump-probe experiments with metals. Appl. Surf. Sci..

[45] Wu C., He M. (2014). Microscopic mechanisms of laser spallation and ablation of metal targets from large-scale molecular dynamics simulations. Appl. Phys. A.

[46] Povarnitsyn M.E., Fokin V.B., Levashov P.R. (2015). Microscopic and macroscopic modeling of femtosecond laser ablation of metals. Appl. Surf. Sci..

[47] Breitling D., Ruf A., Dausinger F. (2004). Fundamental Aspects in Machining of Metals with Short and Ultrashort Laser Pulses. Photon Processing in Microelectronics and Photonics III—Fundamental Aspects in Machining of Metals with Short and Ultrashort Laser Pulses, Proceedings of the SPIE Lasers and Applications in Science and Engineering, San Jose, CA, USA, 25 January 2004.

[48] Lide D.R. (2004). CRC Handbook of Chemistry and Physics.

[49] Wright O.B. (1994). Ultrafast nonequilibrium stress generation in gold and silver. Phys. Rev. B.

[50] Hüttner B., Rohr G. (1996). On the theory of ps and sub-ps laser pulse interaction with metals I. Surface temperature. Appl. Surf. Sci..

[51] Nedialkov N.N., Imamova S.E., Atanasov P.A. (2004). Ablation of metals by ultrashort laser pulses. J. Phys. D Appl. Phys..

[52] Wellershoff S.-S., Hohlfeld J., Güdde J., Matthias E. (1999). The role of electron–phonon coupling in femtosecond laser damage of metals. Appl. Phys. A.

[53] Lin Z., Zhigilei L., Celli V. (2008). Electron-phonon coupling and electron heat capacity of metals under conditions of strong electron-phonon nonequilibrium. Phys. Rev. B.

[54] Foumani A.A., Förster D.J., Ghorbanfekr-Kalashami H., Weber R., Graf T., Niknam A.R. (2021). Atomistic simulation of ultra-short pulsed laser ablation of metals with single and double pulses: An investigation of the re-deposition phenomenon. Appl. Surf. Sci..

[55] Amoruso S., Bruzzese R., Pagano C., Wang X. (2007). Features of plasma plume evolution and material removal efficiency during femtosecond laser ablation of nickel in high vacuum. Appl. Phys. A.

[56] Düsing J.F., Hwang D.J., Grigoropoulos C., Ostendorf A., Kling R. Optical emission imaging and spectroscopy during femtosecond laser ablation of thin metal films on flexible polymer substrates. Proceedings of the International Congress on Applications of Lasers & Electro-Optics.

[57] Förster D.J., Faas S., Gröninger S., Bauer F., Michalowski A., Weber R., Graf T. (2018). Shielding effects and re-deposition of material during processing of metals with bursts of ultra-short laser pulses. Appl. Surf. Sci..

[58] König J., Nolte S., Tünnermann A. (2005). Plasma evolution during metal ablation with ultrashort laser pulses. Opt. Express.

[59] Neuenschwander B., Jaeggi B., Schmid M., Hennig G. (2014). Surface Structuring with Ultra-short Laser Pulses: Basics, Limitations and Needs for High Throughput. Phys. Procedia.

[60] Kramer T., Zhang Y., Remund S., Jaeggi B., Michalowski A., Grad L., Neuenschwander B. (2017). Increasing the Specific Removal Rate for Ultra Short Pulsed Laser-Micromachining by Using Pulse Bursts. JLMN.

[61] Jaeggi B., Cangueiro L., Bruneel D., de Campos J.A.R., Hairaye C., Neuenschwander B. (2018). Micromachining using pulse bursts: Influence of the pulse duration and the number of pulses in the burst on the specific removal rate. Laser Applications in Microelectronic and Optoelectronic Manufacturing (LAMOM) XXIII.

[62] Neuenschwander B., Jaeggi B., Schmid M., Rouffiange V., Martin P.-E. Optimization of the Volume Ablation Rate for Metals at Different Laser Pulse-Durations from ps to fs. Proceedings of the SPIE LASE.

[63] Schille J., Schneider L., Loeschner U. (2015). Process optimization in high-average-power ultrashort pulse laser microfabrication: How laser process parameters influence efficiency, throughput and quality. Appl. Phys. A.

[64] Domke M., Matylitsky V., Stroj S. (2020). Surface ablation efficiency and quality of fs lasers in single-pulse mode, fs lasers in burst mode, and ns lasers. Appl. Surf. Sci..

[65] Raciukaitis G., Brikas M., Gecys P., Voisat B., Gedvilas M. (2009). Use of High Repetition Rate and High Power Lasers in Microfabrication: How to Keep the Efficiency High?. JLMN.

[66] Gillner A., Hartmann C., Dohrn A. (2008). High Quality Micro Machining with Tailored Short and Ultra Short Laser Pulses. Proceedings of the Pacific International Conference on Applications of Lasers and Optics.

[67] Knappe R., Haloui H., Seifert A., Weis A., Nebel A. (2010). Scaling ablation rates for picosecond lasers using burst micromachining. Laser-Based Micro- and Nanopackaging and Assembly IV, Proceedings of the SPIE LASE, San Francisco, CA, USA, 23–28 January 2010.

[68] Vorobyev A.Y., Guo C. (2005). Direct observation of enhanced residual thermal energy coupling to solids in femtosecond laser ablation. Appl. Phys. Lett..

[69] Vorobyev A.Y., Guo C. (2005). Enhanced absorptance of gold following multipulse femtosecond laser ablation. Phys. Rev. B.

[70] Vorobyev A.Y., Guo C. (2006). Enhanced energy coupling in femtosecond laser-metal interactions at high intensities. Opt. Express.

[71] Vorobyev A., Kuzmichev V., Kokody N., Kohns P., Dai J., Guo C. (2005). Residual thermal effects in Al following single ns- and fs-laser pulse ablation. Appl. Phys. A.

[72] Vorobyev A.Y., Guo C. (2011). Thermal response and optical absorptance of metals under femtosecond laser irradiation. Nat. Sci..

[73] Bauer F., Michalowski A., Kiedrowski T., Nolte S. (2015). Heat accumulation in ultra-short pulsed scanning laser ablation of metals. Opt. Express.

[74] Förster D.J., Weber R., Graf T. Residual heat during ultrashort laser drilling of metals. Proceedings of the LPM2017—The 18th International Symposium on Laser Precision Microfabrication.

[75] Bauer F. (2018). Grundlegende Untersuchungen zum Abtragen von Stahl mit Ultrakurzen Laserpulsen. Ph.D. Thesis.

[76] Bornschlegel B., Koller J., Finger J. (2020). In-Situ Analysis of Heat Accumulation during Ultrashort Pulsed Laser Ablation. JLMN.

[77] Wu B., Deng L., Liu P., Zhang F., Duan J., Zeng X. (2017). Effects of picosecond laser repetition rate on ablation of Cr12MoV cold work mold steel. Appl. Surf. Sci..

[78] Metzner D., Lickschat P., Weißmantel S. (2020). Influence of heat accumulation during laser micromachining of CoCrMo alloy with ultrashort pulses in burst mode. Appl. Phys. A.

[79] Martan J., Prokešová L., Moskal D., de Faria B.F., Honner M., Lang V. (2021). Heat accumulation temperature measurement in ultrashort pulse laser micromachining. Int. J. Heat Mass Transf..

[80] Weber R., Graf T., Berger P., Onuseit V., Wiedenmann M., Freitag C., Feuer A. (2014). Heat accumulation during pulsed laser materials processing. Opt. Express.

[81] Weber R., Graf T., Freitag C., Feuer A., Kononenko T., Konov V.I. (2017). Processing constraints resulting from heat accumulation during pulsed and repetitive laser materials processing. Opt. Express.

[82] Di Niso F., Gaudiuso C., Sibillano T., Mezzapesa F.P., Ancona A., Lugarà P.M. (2014). Role of heat accumulation on the incubation effect in multi-shot laser ablation of stainless steel at high repetition rates. Opt. Express.

[83] Raciukaitis G., Brikas M., Gecys P., Gedvilas M. (2008). Accumulation Effects in Laser Ablation of Metals with High-Repetition-Rate Lasers. High-Power Laser Ablation VII, Proceedings of the SPIE LASE, San Francisco, CA, USA, 23–28 January 2008.

[84] Faas S., Bielke U., Weber R., Graf T. (2018). Prediction of the surface structures resulting from heat accumulation during processing with picosecond laser pulses at the average power of 420 W. Appl. Phys. A.

[85] St-Onge L., Sabsabi M., Cielo P. (1998). Analysis of solids using laser-induced plasma spectroscopy in double-pulse mode. Spectrochim. Acta Part B At. Spectrosc..

[86] Mao S., Mao X., Greif R., Russo R.E. (2001). Influence of preformed shock wave on the development of picosecond laser ablation plasma. J. Appl. Phys..

[87] Corsi M., Cristoforetti G., Giuffrida M., Hidalgo M., Legnaioli S., Palleschi V., Salvetti A., Tognoni E., Vallebona C. (2004). Three-dimensional analysis of laser induced plasmas in single and double pulse configuration. Spectrochim. Acta Part B At. Spectrosc..

[88] Scuderi D., Albert O., Moreau D., Pronko P.P., Etchepare J. (2005). Interaction of a laser-produced plume with a second time delayed femtosecond pulse. Appl. Phys. Lett..

[89] Hanada Y., Sugioka K., Miyamoto I., Midorikawa K. (2005). Double-pulse irradiation by laser-induced plasma-assisted ablation (LIPAA) and mechanisms study. Appl. Surf. Sci..

[90] Le Harzic R., Breitling D., Sommer S., Föhl C., König K., Dausinger F., Audouard E. (2005). Processing of metals by double pulses with short laser pulses. Appl. Phys. A.

[91] Babushok V., DeLucia F., Gottfried J., Munson C., Miziolek A. (2006). Double pulse laser ablation and plasma: Laser induced breakdown spectroscopy signal enhancement. Spectrochim. Acta Part B At. Spectrosc..

[92] Suttmann O., Wojakowski B., Klug U., Kling R., Ostendorf A. (2008). Picosecond double-pulse ablation in silicon and aluminium with variable delay. J. Laser Appl..

[93] Bogaerts A., Chen Z., Autrique D. (2008). Double pulse laser ablation and laser induced breakdown spectroscopy: A modeling investigation. Spectrochim. Acta Part B At. Spectrosc..

[94] Singha S., Hu Z., Gordon R.J. (2008). Ablation and plasma emission produced by dual femtosecond laser pulses. J. Appl. Phys..

[95] Noel S., Hermann J. (2009). Reducing nanoparticles in metal ablation plumes produced by two delayed short laser pulses. Appl. Phys. Lett..

[96] Wojakowski B., Suttmann O., Klug U., Kling R. (2009). Micromachining with Picosecond Double Pulses on Silicon and Aluminium. Laser-Based Micro- and Nanopackaging and Assembly III, Proceedings of the SPIE LASE: Lasers and Applications in Science and Engineering, San Jose, CA, USA, 24 January 2009.

[97] Roberts D., du Plessis A., Botha L. (2010). Femtosecond laser ablation of silver foil with single and double pulses. Appl. Surf. Sci..

[98] Deladurantaye P., Cournoyer A., Drolet M., Desbiens L., Lemieux D., Briand M., Taillon Y. (2011). Material Micromachining Using Bursts of High Repetition Rate Picosecond Pulses from a Fiber Laser Source. Fiber Lasers VIII: Technology, Systems, and Applications, Proceedings of the SPIE LASE San Francisco, CA, USA, 22–27 January 2011.

[99] Axente E., Mihailescu I.N., Itina T., Hermann J. (2011). Probing electron-phonon coupling in metals via observations of ablation plumes produced by two delayed short laser pulses. Appl. Phys. Lett..

[100] Sailer M., Bauer F., Kleiner J., Kaiser M. Scaling of ablation rates. Ablation efficiency and quality aspects of “Burstmode”—Micromachining of metals. Proceedings of the Lasers in Manufacturing Conference.

[101] Hänel N., Stolze M., Herrmann T.R.W., Lhuillier J.A. (2015). Fundamental Investigations of ps-Laser Burst-Mode on Common Metals for an Enhanced Ablation Process. Laser-Based Micro- and Nanoprocessing IX, Proceedings of the SPIE LASE San Francisco, CA, USA, 7–12 February 2015.

[102] Kramer T., Neuenschwander B., Jäggi B., Remund S., Hunziker U., Zürcher J. (2016). Influence of Pulse Bursts on the Specific Removal Rate for Ultra-fast Pulsed Laser Micromachining of Copper. Phys. Procedia.

[103] Schille J., Schneider L., Kraft S., Hartwig L., Loeschner U. (2016). Experimental study on double-pulse laser ablation of steel upon multiple parallel-polarized ultrashort-pulse irradiations. Appl. Phys. A.

[104] Finger J. (2017). Puls-zu-Puls-Wechselwirkungen beim Ultrakurzpuls-Laserabtrag mit hohen Repetitionsraten. Ph.D. Thesis.

[105] Lickschat P., Demba A., Weissmantel S. (2017). Ablation of steel using picosecond laser pulses in burst mode. Appl. Phys. A.

[106] Mayerhofer R. (2017). Ultrashort-pulsed laser material processing with high repetition rate burst pulses. Laser Applications in Microelectronic and Optoelectronic Manufacturing (LAMOM) XXII, Proceedings of the SPIE LASE, 2017, San Francisco, CA, USA, 28 January–2 February 2017.

[107] Jaeggi B., Remund S., Zhang Y., Kramer T., Neuenschwander B. (2017). Optimizing the Specific Removal Rate with the Burst Mode Under Varying Conditions. JLMN.

[108] Rosenfeld A., Höhm S., Krüger J., Bonse J. (2018). Dynamics of Ultrashort Double-Pulse Laser Ablation of Solid Surfaces. Encyclopedia of Interfacial Chemistry.

[109] Hashida M., Masuno S., Furukawa Y., Kusaba M., Inoue S., Sakabe S., Sakagami H., Tsukamoto M. (2018). Suppression of ablation by double-pulse femtosecond laser irradiation. Frontiers in Ultrafast Optics: Biomedical, Scientific, and Industrial Applications XVIII, Proceedings of the SPIE LASE, San Francisco, CA, USA, 27 January–1 February 2018.

[110] Zhang K., Zhang J., Jiang L., Li X., Liu Y., Li B., Lu Y. (2018). Ablation enhancement of metal in ultrashort double-pulse experiments. Appl. Phys. Lett..

[111] Takenaka K., Tsukamoto M., Hashida M., Masuno S., Sakagami H., Kusaba M., Sakabe S., Inoue S., Furukawa Y., Asai S. (2019). Ablation suppression of a titanium surface interacting with a two-color double-pulse femtosecond laser beam. Appl. Surf. Sci..

[112] Bruening S., Du K., Gillner A. (2020). Micro processing with ultrafast bursts of pulses. Procedia CIRP.

[113] Lin Z., Ji L., Hong M. (2020). Enhancement of femtosecond laser-induced surface ablation via temporal overlapping double-pulse irradiation. Photon Res..

[114] Förster D.J., Faas S., Weber R., Graf T. (2020). Thrust enhancement and propellant conservation for laser propulsion using ultra-short double pulses. Appl. Surf. Sci..

[115] Cheng C.-W., Chen J.-K. (2016). Drilling of Copper Using a Dual-Pulse Femtosecond Laser. Technologies.

[116] Förster G.D., Lewis L.J. (2018). Numerical study of double-pulse laser ablation of Al. Phys. Rev. B.

[117] Roth J., Krauß A., Lotze J., Trebin H.-R. (2014). Simulation of laser ablation in aluminum: The effectivity of double pulses. Appl. Phys. A.

[118] Povarnitsyn M.E., Itina T.E., Levashov P.R., Khishchenko K. (2011). Simulation of ultrashort double-pulse laser ablation. Appl. Surf. Sci..

[119] Kudryashov S.I., Samokhvalov A.A., Golubev Y.D., Ivanov D.S., Garcia M.E., Veiko V.P., Rethfeld B., Mikhailovskii V.Y. (2021). Dynamic all-optical control in ultrashort double-pulse laser ablation. Appl. Surf. Sci..

[120] Spellauge M., Winter J., Rapp S., McDonnell C., Sotier F., Schmidt M., Huber H.P. (2021). Influence of stress confinement, particle shielding and re-deposition on the ultrashort pulse laser ablation of metals revealed by ultrafast time-resolved experiments. Appl. Surf. Sci..

[121] Ackerl N. (2020). Laser Surface Functionalization from Fundamentals to Application. Ph.D. Thesis.

[122] Börner P. (2019). Ultra-Short Pulsed Laser Ablation of Diamond. Ph.D. Thesis.

[123] Furukawa Y., Inoue S., Hashida M. (2021). Temporal change in laser penetration length of titanium and platinum for double-pulse ablation measured by a novel ablation method. J. Laser Appl..

[124] Lauer B., Jaeggi B., Zhang Y., Neuenschwander B. (2015). Measurement of the maximum specific removal rate: Unexpected influence of the experimental method and the spot size. Proceedings of the International Congress on Applications of Lasers & Electro-Optics.

[125] Kramer T., Remund S., Jäggi B., Schmid M., Neuenschwander B. (2018). Ablation dynamics—From absorption to heat accumulation/ultra-fast laser matter interaction. Adv. Opt. Technol..

[126] Winter J., Rapp S., Schmidt M., Huber H.P. (2017). Ultrafast laser processing of copper: A comparative study of experimental and simulated transient optical properties. Appl. Surf. Sci..

[127] Neuenschwander B., Bucher G.F., Nussbaum C., Joss B., Muralt M., Hunziker U.W., Schuetz P. (2010). Processing of metals and dielectric materials with ps-laser pulses: Results, strategies, limitations and needs. Laser Applications in Microelectronic and Optoelectronic Manufacturing XV, Proceedings of the SPIE LASE, San Francisco, CA, USA, 12 March 2010.

[128] Osbild M., Brenner A., Röther L., Finger J. (2020). Ultrashort pulse laser micro polishing of steel—Investigation of the melt pool depth. Procedia CIRP.

[129] Remund S., Kramer T., Neuenschwander B., Jäggi B. (2020). Method for Producing an Implant, and Implant Produced by Said Method. U.S. Patent.

[130] Brenner A., Röther L., Osbild M., Finger J. (2020). Laser Polishing Using Ultrashort Pulse Laser. Laser-Based Micro- and Nanoprocessing XIV, Proceedings of the SPIE LASE, San Francisco, CA, USA, 1–6 February 2020.

[131] Metzner D., Lickschat P., Weißmantel S. (2021). Optimization of the ablation process using ultrashort pulsed laser radiation in different burst modes. J. Laser Appl..

[132] Metzner D., Lickschat P., Weißmantel S. (2021). Surface treatment on cobalt and titanium alloys using picosecond laser pulses in burst mode. Appl. Phys. A.

[133] Matsumoto H., Lin Z., Kleinert J. (2018). Ultrafast Laser Ablation of Copper with ~GHz Bursts. Laser Applications in Microelectronic and Optoelectronic Manufacturing (LAMOM) XXIII (International Society for Optics and Photonics, Proceedings of the SPIE LASE, San Francisco, CA, USA, 29–31 January 2018.

[134] Povarnitsyn M.E., Levashov P.R., Knyazev D.V. (2018). Simulation of ultrafast bursts of subpicosecond pulses: In pursuit of efficiency. Appl. Phys. Lett..

[135] Bonamis G., Audouard E., Hoenninger C., Lopez J., Mishchik K., Mottay E., Manek-Hönninger I. (2020). Systematic study of laser ablation with GHz bursts of femtosecond pulses. Opt. Express.

[136] Bonamis G., Mishchick K., Audouard E., Hönninger C., Mottay E., Lopez J., Manek-Hönninger I. (2019). High efficiency femtosecond laser ablation with gigahertz level bursts. J. Laser Appl..

[137] Butkus S., Jukna V., Paipulas D., Barkauskas M., Sirutkaitis V. (2020). Micromachining of Invar Foils with GHz, MHz and kHz Femtosecond Burst Modes. Micromachines.

[138] Hendow S.T., Takahashi H., Yamaguchi M., Xu J. (2020). Enhanced Ablation Using GHz-Pulsed fs Laser. Laser-Based Micro- and Nanoprocessing XIV, Proceedings of the SPIE LASE, San Francisco, CA, USA, 1–6 February 2020.

[139] Hirsiger T., Gafner M., Remund S.M., Chaja M.W., Urniezius A., Butkus S., Neuenschwander B. (2020). Machining Metals and Silicon with GHz Bursts: Surprising Tremendous Reduction of the Specific Removal Rate for Surface Texturing Applications. Laser Applications in Microelectronic and Optoelectronic Manufacturing (LAMOM) XXV, Proceedings of the SPIE LASE, San Francisco, CA, USA, 1–6 February 2020.

[140] Cheng C.-W., Chen J.-K. (2020). Ultrafast laser ablation of copper by GHz bursts. Appl. Phys. A.

[141] Fehrenbacher A., Sailer M., Fuehra B., Jansen F., Tan C., Baumbach S., Flaig R., Eberhardt C., Ruebling S., Quentin U. (2021). New generation TruMicro Series 2000: Micromachining Versatility by GHz-burst, Higher Average Power, Flexible Pulse on Demand and Integrated Hollow-Core Fiber Interface. Laser-Based Micro- and Nanoprocessing XV, Proceedings of the SPIE LASE, San Francisco, CA, USA, 6–11 March 2021.

[142] Nyenhuis F., Michalowski A., L’Huillier J.A. (2020). Surface Treatment with GHz-Bursts. Laser-Based Micro- and Nanoprocessing XIV, Proceedings of the SPIE LASE, San Francisco, CA, USA, 1–6 February 2020.

[143] Michalowski A., Nyenhuis F., Kunz G. (2020). Smooth Surfaces by Pulsed Laser Processing with Bursts. Photonics Views.

[144] Obata K., Caballero-Lucas F., Sugioka K. (2021). Material Processing at GHz Burst Mode by Femtosecond Laser Ablation. JLMN.

[145] Manninen M., Hirvimäki M., Poutiainen I., Salminen A. (2015). Effect of Pulse Length on Engraving Efficiency in Nanosecond Pulsed Laser Engraving of Stainless Steel. Met. Mater. Trans. A.

[146] Mathew M.M., Bathe R.N., Padmanabham G., Padmanaban R., Thirumalini S. (2017). A study on the micromachining of molybdenum using nanosecond and femtosecond lasers. Int. J. Adv. Manuf. Technol..

[147] Garnov S., Konov V., Kononenko T., Pashinin V., Sinyavsky M. (2004). Microsecond laser material processing at 1.06 µm. Laser Phys..

[148] Mur J., Petkovšek R. (2018). Precision and resolution in laser direct microstructuring with bursts of picosecond pulses. Appl. Phys. A.

[149] Gaudiuso C., Giannuzzi G., Volpe A., Lugarà P.M., Choquet I., Ancona A. (2018). Incubation during laser ablation with bursts of femtosecond pulses with picosecond delays. Opt. Express.

[150] Rong Y., Ji P., He M., Zhang Y., Tang Y. (2018). Multiscale Investigation of Femtosecond Laser Pulses Processing Aluminum in Burst Mode. Nanoscale Microscale Thermophys. Eng..

[151] Mur J., Petkovšek R. (2019). Near-THz bursts of pulses—Governing surface ablation mechanisms for laser material processing. Appl. Surf. Sci..

[152] Wang A., Das A., Grojo D. (2020). Ultrafast Laser Writing Deep inside Silicon with THz-Repetition-Rate Trains of Pulses. Research.

[153] Ancona A., Gaudiuso C., Giannuzzi G., Choquet I., Lugarà P.M. (2018). Incubation Effect In Burst Mode Fs-Laser Ablation Of Stainless Steel Samples. Laser-Based Micro- and Nanoprocessing XII, Proceedings of the SPIE LASE, San Francisco, CA, USA, 27 January–1 February 2020.

[154] Förster D.J., Weber R., Holder D., Graf T. (2018). Estimation of the depth limit for percussion drilling with picosecond laser pulses. Opt. Express.

[155] Holder D., Weber R., Graf T., Onuseit V., Brinkmeier D., Förster D.J., Feuer A. (2021). Analytical model for the depth progress of percussion drilling with ultrashort laser pulses. Appl. Phys. A.

[156] Lapczyna M., Chen K., Herman P., Tan H., Marjoribanks R. (1999). Ultra high repetition rate (133 MHz) laser ablation of aluminum with 1.2-ps pulses. Appl. Phys. A.

[157] Esser D., Rezaei S., Li J., Herman P.R., Gottmann J. (2011). Time dynamics of burst-train filamentation assisted femtosecond laser machining in glasses. Opt. Express.

[158] Kammer H., Dreisow F., Tünnermann A. (2016). Analysis of the Hole Shape Evolution in fs-Pulse Percussion Drilling with Bursts. Frontiers in Ultrafast Optics: Biomedical, Scientific, and Industrial Applications XVI, Proceedings of the SPIE LASE, San Francisco, CA, USA, 13–18 February 2016.

[159] Wang Q., Luo S., Chen Z., Qi H., Deng J., Hu Z. (2016). Drilling of aluminum and copper films with femtosecond double-pulse laser. Opt. Laser Technol..

[160] Balachninaitė O., Tamulienė V., Eičas L., Vaičaitis V. (2021). Laser micromachining of steel and copper using femtosecond laser pulses in GHz burst mode. Results Phys..

[161] Schille J., Loeschner U., Ebert R., Scully P., Goddard N., Exner H. (2010). Laser micro processing using a high repetition rate femto second laser. J. Laser Appl..

[162] Tsukamoto M., Kayahara T., Nakano H., Hashida M., Katto M., Fujita M., Tanaka M., Abe N. (2007). Microstructures formation on titanium plate by femtosecond laser ablation. J. Physics Conf. Ser..

[163] Herrmann T., Harth F., Henrich B., L’Huillier J., Hajri M. (2016). How to Improve Efficiency in USP Laser Micromachining. Laser Tech. J..

[164] Nyenhuis F., Michalowski A., L’Huillier J. (2020). Dual Process Strategy to Increase the Usable Power for Laser-Milling. JLMN.

[165] Sassmannshausen A., Brenner A., Finger J. (2021). Ultrashort pulse laser polishing by continuous surface melting. J. Mater. Process. Technol..

[166] Kažukauskas E., Butkus S., Tokarski P., Jukna V., Barkauskas M., Sirutkaitis V. (2020). Micromachining of Transparent Biocompatible Polymers Applied in Medicine Using Bursts of Femtosecond Laser Pulses. Micromachines.

[167] Antończak A.J., Stępak B., Kozioł P.E., Abramski K.M. (2013). The influence of process parameters on the laser-induced coloring of titanium. Appl. Phys. A.

[168] Veiko V., Odintsova G., Ageev E., Karlagina Y., Loginov A., Skuratova A., Gorbunova E. (2014). Controlled oxide films formation by nanosecond laser pulses for color marking. Opt. Express.

[169] Lecka K.M., Wojcik M.R., Antonczak A.J. (2017). Laser-Induced Color Marking of Titanium: A Modeling Study of the Interference Effect and the Impact of Protective Coating. Math. Probl. Eng..

[170] Liu H., Lin W., Hong M. (2019). Surface coloring by laser irradiation of solid substrates. APL Photon..

[171] Vorobyev A.Y., Guo C. (2008). Colorizing metals with femtosecond laser pulses. Appl. Phys. Lett..

[172] Dusser B., Sagan S., Soder H., Faure N., Colombier J.-P., Jourlin M., Audouard E. (2010). Controlled nanostructrures formation by ultra fast laser pulses for color marking. Opt. Express.

[173] Ackerl N., Gugger P., Warhanek M., Gysel J., Wegener K. (2020). Ultra-Short Pulsed Laser Marking and Coloration of Metals with Segmented Pixel Parameter Transformation. JLMN.

[174] Vorobyev A.Y., Guo C. (2013). Direct femtosecond laser surface nano/microstructuring and its applications: Direct femtosecond laser surface nano/microstructuring and its applications. Laser Photonics Rev..

[175] Guay J.-M., Lesina A.C., Cote G., Charron M., Poitras D., Ramunno L., Berini P., Weck A. (2017). Laser-induced plasmonic colours on metals. Nat. Commun..

[176] Berini P., Guay J.-M., Lesina A.C., Walia J., Ramunno L., Weck A., Krupin O., Warren M. (2018). Plasmonic Colours on Bulk Metals: Laser Coloring of Large Areas Exhibiting High Topography. Frontiers in Ultrafast Optics: Biomedical, Scientific, and Industrial Applications XVIII, Proceedings of the SPIE LASE, San Francisco, CA, USA, 27 January–1 February 2018.

[177] Lumentum (2016). FlexBurstTM—Picosecond Micromachining Lasers Offer “Burst Mode” with Full User Control of Intra-Burst Pulse Energy Distribution.

[178] Guay J.-M., Lesina A.C., Baxter J., Killaire G., Ramunno L., Berini P., Weck A. (2018). Topography Tuning for Plasmonic Color Enhancement via Picosecond Laser Bursts. Adv. Opt. Mater..

[179] Stratakis E., Bonse J., Heitz J., Siegel J., Tsibidis G., Skoulas E., Papadopoulos A., Mimidis A., Joel A.-C., Comanns P. (2020). Laser engineering of biomimetic surfaces. Mater. Sci. Eng. R Rep..

[180] Masato D., Sorgato M., Batal A., Dimov S., Lucchetta G. (2019). Thin-wall injection molding of polypropylene using molds with different laser-induced periodic surface structures. Polym. Eng. Sci..

[181] Florian C., Kirner S.V., Krüger J., Bonse J. (2020). Surface functionalization by laser-induced periodic surface structures. J. Laser Appl..

[182] Piccolo L., Sorgato M., Batal A., Dimov S., Lucchetta G., Masato D. (2020). Functionalization of Plastic Parts by Replication of Variable Pitch Laser-Induced Periodic Surface Structures. Micromachines.

[183] Bonse J., Höhm S., Kirner S.V., Rosenfeld A., Krüger J. (2017). Laser-Induced Periodic Surface Structures—A Scientific Evergreen. IEEE J. Sel. Top. Quantum Electron..

[184] Bonse J., Graf S. (2020). Maxwell Meets Marangoni—A Review of Theories on Laser-Induced Periodic Surface Structures. Laser Photonics Rev..

[185] Bonse J. (2020). Quo Vadis LIPSS? Recent and Future Trends on Laser-Induced Periodic Surface Structures. Nanomaterials.

[186] Bonse J., Kirner S.V., Krüger J., Sugioka K. (2021). Laser-Induced Periodic Surface Structures (LIPSS). Handbook of Laser Micro- and Nano-Engineering.

[187] Fraggelakis F., Mincuzzi G., Lopez J., Manek-Hönninger I., Kling R. (2019). Controlling 2D laser nano structuring over large area with double femtosecond pulses—ScienceDirect. Appl. Surf. Sci..

[188] Hashida M., Furukawa Y., Inoue S., Sakabe S., Masuno S., Kusaba M., Sakagami H., Tsukamoto M. (2020). Uniform LIPSS on titanium irradiated by two-color double-pulse beam of femtosecond laser. J. Laser Appl..

[189] Hashida M. (2014). Periodic Grating Structures on Metal Self-organized by Double-pulse Irradiation. J. Laser Micro Nanoeng..

[190] Hashida M., Nishii T., Miyasaka Y., Sakagami H., Shimizu M., Inoue S., Sakabe S. (2016). Orientation of periodic grating structures controlled by double-pulse irradiation. Appl. Phys. A.

[191] Giannuzzi G., Gaudiuso C., Di Franco C., Scamarcio G., Lugarà P.M., Ancona A. (2019). Large area laser-induced periodic surface structures on steel by bursts of femtosecond pulses with picosecond delays. Opt. Lasers Eng..

[192] Giannuzzi G., Gaudiuso C., Cinquino M., Di Mundo R., Mirenghi L., Di Franco C., Scamarcio G., Lugarà P.M., Ancona A. (2019). 1-D and 2-D Surface Structuring of Steel by Bursts of Femtosecond Laser Pulses. Laser-Based Micro- and Nanoprocessing XIII, Proceedings of the SPIE LASE, San Francisco, CA, USA, 4 February 2019.

[193] Wang X., Li C., Ma C., Feng J., Hong W., Zhang Z. (2018). Formation of laser induced periodic structures on stainless steel using multi-burst picosecond pulses. Opt. Express.

[194] Fraggelakis F., Giannuzzi G., Gaudiuso C., Manek-Hönninger I., Mincuzzi G., Ancona A., Kling R. (2019). Double- and Multi-Femtosecond Pulses Produced by Birefringent Crystals for the Generation of 2D Laser-Induced Structures on a Stainless Steel Surface. Materials.

[195] Höhm S., Rosenfeld A., Krüger J., Bonse J. (2013). Area dependence of femtosecond laser-induced periodic surface structures for varying band gap materials after double pulse excitation. Appl. Surf. Sci..

[196] Höhm S., Herzlieb M., Rosenfeld A., Krüger J., Bonse J. (2015). Femtosecond laser-induced periodic surface structures on silicon upon polarization controlled two-color double-pulse irradiation. Opt. Express.

[197] Jaeggi B., Neuenschwander B., Zimmermann M., Zecherle M., Boeckler E.W. (2016). Time-Optimized Laser Micro Machining by Using a New High Dynamic and High Precision Galvo Scanner. Laser Applications in Microelectronic and Optoelectronic Manufacturing (LAMOM) XXI, Proceedings of the SPIE LASE, San Francisco, CA, USA, 13–18 February 2016.

[198] Häfner T., Heberle J., Holder D., Schmidt M. Adjustment of surface energy on steel surfaces due to CLP generation by picosecond laser processing. Proceedings of the Lasers in Manufacturing Conference.

[199] Jaeggi B., Neuenschwander B., Hunziker U., Zuercher J., Meier T., Zimmermann M., Selbmann K.H., Hennig G. (2012). Ultra-High-Precision Surface Structuring by Synchronizing a Galvo Scanner with an Ultra-Short-Pulsed Laser System in MOPA Arrangement. Laser Applications in Microelectronic and Optoelectronic Manufacturing (LAMOM) XVII, Proceedings of the SPIE LASE, San Francisco, CA, USA, 21–26 January 2012.

[200] Loeschner U., Schille J., Streek A., Knebel T., Hartwig L., Hillmann R., Endisch C. (2015). High-rate laser microprocessing using a polygon scanner system. J. Laser Appl..

[201] Schille J., Schneider L., Mauersberger S., Szokup S., Höhn S., Pötschke J., Reiß F., Leidich E., Löschner U. (2020). High-Rate Laser Surface Texturing for Advanced Tribological Functionality. Lubricants.

[202] Jaeggi B., Remund S., Streubel R., Goekce B., Barcikowski S., Neuenschwander B. (2017). Laser Micromachining of Metals with Ultra-Short Pulses: Factors Limiting the Scale-Up Process. JLMN.

[203] Brenner A., Zecherle M., Verpoort S., Schuster K., Schnitzler C., Kogel-Hollacher M., Reisacher M., Nohn B. (2020). Efficient production of design textures on large-format 3D mold tools. J. Laser Appl..

[204] Phipps C., Luke J. (2002). Diode Laser-Driven Microthrusters: A New Departure for Micropropulsion. AIAA J..

[205] Phipps C., Birkan M., Bohn W., Eckel H.-A., Horisawa H., Lippert T., Michaelis M., Rezunkov Y., Sasoh A., Schall W. (2010). Review: Laser-Ablation Propulsion. J. Propuls. Power.

[206] Phipps C.R., Baker K.L., Libby S., Liedahl D.A., Olivier S.S., Pleasance L.D., Rubenchik A., Trebes J.E., George E.V., Marcovici B. (2012). Removing orbital debris with lasers. Adv. Space Res..

[207] Scharring S., Lorbeer R.A., Karg S., Pastuschka L., Förster D.J., Eckel H.A. (2016). The MICROLAS Concept: Precise Thrust Generation in the Micronewton Range by Laser Ablation. Technology for Small Satellite Research: Payloads and Subsystem Technologies Small Satellite Applications, Missions, and In-Orbit Experiences.

[208] Phipps C.R. (2018). Laser Ablation Propulsion and Its Applications in Space. Superconductivity.

[209] Levchenko I., Bazaka K., Mazouffre S., Xu S. (2018). Prospects and physical mechanisms for photonic space propulsion. Nat. Photon..

[210] Lorbeer R.-A., Zwilich M., Zabic M., Scharring S., Eisert L., Wilken J., Schumacher D., Roth M., Eckel H.-A. (2018). Experimental verification of high energy laser-generated impulse for remote laser control of space debris. Sci. Rep..

[211] Cremers D.A., Knight A.K., Meyers R.A. (2000). Laser-Induced Breakdown Spectroscopy. Encyclopedia of Analytical Chemistry.

[212] Song K., Lee Y.-I., Sneddon J. (2002). Recent developments in instrumentation for laser induced breakdown spectroscopy. Appl. Spectrosc. Rev..

[213] Miziolek A.W., Palleschi V., Schechter I. (2006). Laser-Induced Breakdown Spectroscopy (LIBS): Fundamentals and Applications.

[214] Kim B.-M., Feit M., Rubenchik A., Mammini B., Da Silva L. (1998). Optical feedback signal for ultrashort laser pulse ablation of tissue. Appl. Surf. Sci..

[215] Margetic V., Pakulev A., Stockhaus A., Bolshov M., Niemax K., Hergenröder R. (2000). A comparison of nanosecond and femtosecond laser-induced plasma spectroscopy of brass samples. Spectrochim. Acta Part B At. Spectrosc..

[216] Burgio L., Melessanaki K., Doulgeridis M., Clark R., Anglos D. (2001). Pigment identification in paintings employing laser induced breakdown spectroscopy and Raman microscopy. Spectrochim. Acta Part B At. Spectrosc..

[217] Serifaki K., Boke H., Yalcin S., Ipekoglu B. (2009). Characterization of materials used in the execution of historic oil paintings by XRD, SEM-EDS, TGA and LIBS analysis. Mater. Charact..

[218] Borba F.D.S.L., Cortez J., Asfora V.K., Pasquini C., Pimentel M.F., Pessis A.-M., Khoury H.J. (2012). Multivariate treatment of LIBS data of prehistoric paintings. J. Braz. Chem. Soc..

[219] Dikmelik Y., McEnnis C., Spicer J.B. (2008). Femtosecond and nanosecond laser-induced breakdown spectroscopy of trinitrotoluene. Opt. Express.

[220] Gottfried J.L., De Lucia F.C., Munson C.A., Miziolek A.W. (2009). Laser-induced breakdown spectroscopy for detection of explosives residues: A review of recent advances, challenges, and future prospects. Anal. Bioanal. Chem..

[221] Shaik A.K., Epuru N.R., Syed H., Byram C., Soma V.R. (2018). Femtosecond laser induced breakdown spectroscopy based standoff detection of explosives and discrimination using principal component analysis. Opt. Express.

[222] Gurevich E.L., Hergenröder R. (2007). Femtosecond Laser-Induced Breakdown Spectroscopy: Physics, Applications, and Perspectives. Appl. Spectrosc..

[223] Malvezzi A.M., Musazzi S., Perini U. (2014). Laser–Matter Interaction in LIBS Experiments. Laser-Induced Breakdown Spectroscopy.

[224] Labutin T.A., Lednev V.N., Ilyin A.A., Popov A. (2015). Femtosecond laser-induced breakdown spectroscopy. J. Anal. At. Spectrom..

[225] Piñon V., Fotakis C., Nicolas G., Anglos D. (2008). Double pulse laser-induced breakdown spectroscopy with femtosecond laser pulses. Spectrochim. Acta Part B At. Spectrosc..

[226] Lu Y., Zorba V., Mao X., Zheng R., Russo R.E. (2013). UV fs–ns double-pulse laser induced breakdown spectroscopy for high spatial resolution chemical analysis. J. Anal. At. Spectrom..

[227] Bulanov S.V., Khoroshkov V.S. (2002). Feasibility of using laser ion accelerators in proton therapy. Plasma Phys. Rep..

[228] Ditmire T., Donnelly T., Rubenchik A.M., Falcone R.W., Perry M.D. (1996). Interaction of intense laser pulses with atomic clusters. Phys. Rev. A.

[229] Malka V., Fritzler S. (2004). Electron and proton beams produced by ultra short laser pulses in the relativistic regime. Laser Part. Beams.

[230] Neely D., Foster P., Robinson A., Lindau F., Lundh O., Persson A., Wahlström C.-G., McKenna P. (2006). Enhanced proton beams from ultrathin targets driven by high contrast laser pulses. Appl. Phys. Lett..

[231] Andreev A., Sonobe R., Kawata S., Miyazaki S., Sakai K., Miyauchi K., Kikuchi T., Platonov K., Nemoto K. (2006). Effect of a laser prepulse on fast ion generation in the interaction of ultra-short intense laser pulses with a limited-mass foil target. Plasma Phys. Control. Fusion.

[232] Passoni M., Bertagna L., Zani A. (2010). Target normal sheath acceleration: Theory, comparison with experiments and future perspectives. New J. Phys..

[233] Daido H., Nishiuchi M., Pirozhkov A. (2012). Review of laser-driven ion sources and their applications. Rep. Prog. Phys..

[234] Fourmaux S., Buffechoux S., Albertazzi B., Capelli D., Levy A., Gnedyuk S., Lecherbourg L., Lassonde P., Payeur S., Antici P. (2013). Investigation of laser-driven proton acceleration using ultra-short, ultra-intense laser pulses. Phys. Plasmas.

[235] Barberio M., Veltri S., Scisciò M., Antici P. (2017). Laser-Accelerated Proton Beams as Diagnostics for Cultural Heritage. Sci. Rep..

[236] Barberio M., Scisciò M., Vallières S., Cardelli F., Chen S.N., Famulari G., Gangolf T., Revet G., Schiavi A., Senzacqua M. (2018). Laser-accelerated particle beams for stress testing of materials. Nat. Commun..

[237] Torrisi L., Cutroneo M., Torrisi A., Silipigni L., Costa G., Rosinski M., Badziak J., Wołowski J., Zaraś-Szydłowska A., Parys P. (2019). Protons accelerated in the target normal sheath acceleration regime by a femtosecond laser. Phys. Rev. Accel. Beams.

[238] Bulanov S., Esirkepov T., Khoroshkov V., Kuznetsov A., Pegoraro F. (2002). Oncological hadrontherapy with laser ion accelerators. Phys. Lett. A.

[239] Malka V., Faure J., Gauduel Y.A. (2010). Ultra-short electron beams based spatio-temporal radiation biology and radiotherapy. Mutat. Res. Mutat. Res..

[240] Schardt D., Elsaesser T., Schulz-Ertner D. (2010). Heavy-ion tumor therapy: Physical and radiobiological benefits. Rev. Mod. Phys..

[241] Döppner T., Fennel T., Diederich T., Tiggesbäumker J., Meiwes-Broer K.H. (2005). Controlling the Coulomb Explosion of Silver Clusters by Femtosecond Dual-Pulse Laser Excitation. Phys. Rev. Lett..

[242] Markey K., McKenna P., Brenner C.M., Carroll D.C., Günther M.M., Harres K., Kar S., Lancaster K., Nürnberg F., Quinn M.N. (2010). Spectral Enhancement in the Double Pulse Regime of Laser Proton Acceleration. Phys. Rev. Lett..

[243] Uryupina D.S., Ivanov K.A., Brantov A., Savel’Ev A.B., Bychenkov V.Y., Povarnitsyn M.E., Volkov R.V., Tikhonchuk V.T. (2012). Femtosecond laser-plasma interaction with prepulse-generated liquid metal microjets. Phys. Plasmas.

